# The link between amyloid β and ferroptosis pathway in Alzheimer’s disease progression

**DOI:** 10.1038/s41419-024-07152-0

**Published:** 2024-10-28

**Authors:** Naďa Majerníková, Alejandro Marmolejo-Garza, Casandra Salinas Salinas, Minh D. A. Luu, Yuequ Zhang, Marina Trombetta-Lima, Tamara Tomin, Ruth Birner-Gruenberger, Šárka Lehtonen, Jari Koistinaho, Justina C. Wolters, Scott Ayton, Wilfred F. A. den Dunnen, Amalia M. Dolga

**Affiliations:** 1 Department of Molecular Pharmacology, Groningen Research Institute of Pharmacy, Research School of Behavioural and Cognitive Neuroscience, University of Groningen, Groningen, The Netherlands; 2 Department of Pathology and Medical Biology, Research Institute Brain and Cognition, Molecular Neuroscience and Aging Research, Research School of Behavioural and Cognitive Neuroscience, University Medical Centre Groningen, University of Groningen, Groningen, The Netherlands; 3 Department of Biomedical Sciences of Cells and Systems, Molecular Neurobiology Section, University of Groningen, University Medical Center, Groningen, The Netherlands; 4 Department of Pharmaceutical Technology and Biopharmacy, Groningen Research Institute of Pharmacy, University of Groningen, Groningen, The Netherlands; 5 Institute of Chemical Technologies and Analytics, Faculty of Technical Chemistry, Technische Universität Wien, Vienna, Austria; 6 A. I. Virtanen Institute for Molecular Sciences, University of Eastern Finland, Kuopio, Finland; 7 Neuroscience Center, HiLIFE, University of Helsinki, Helsinki, Finland; 8 Laboratory of Pediatrics, Section Systems Medicine of Metabolism and Signaling, Faculty of Medical Sciences, University of Groningen, University Medical Center Groningen, Groningen, The Netherlands; 9 The Florey Neuroscience Institute, The University of Melbourne, Parkville, VIC Australia

**Keywords:** Cell death in the nervous system, Alzheimer's disease

## Abstract

Alzheimer’s disease (AD) affects millions of people worldwide and represents the most prevalent form of dementia. Treatment strategies aiming to interfere with the formation of amyloid β (Aβ) plaques and neurofibrillary tangles (NFTs), the two major AD hallmarks, have shown modest or no effect. Recent evidence suggests that ferroptosis, a type of programmed cell death caused by iron accumulation and lipid peroxidation, contributes to AD pathogenesis. The existing link between ferroptosis and AD has been largely based on cell culture and animal studies, while evidence from human brain tissue is limited. Here we evaluate if Aβ is associated with ferroptosis pathways in post-mortem human brain tissue and whether ferroptosis inhibition could attenuate Aβ-related effects in human brain organoids. Performing positive pixel density scoring on immunohistochemically stained post-mortem Brodmann Area 17 sections revealed that the progression of AD pathology was accompanied by decreased expression of nuclear receptor co-activator 4 and glutathione peroxidase 4 in the grey matter. Differentiating between white and grey matter, allowed for a more precise understanding of the disease’s impact on different brain regions. In addition, ferroptosis inhibition prevented Aβ pathology, decreased lipid peroxidation and restored iron storage in human AD iPSCs-derived brain cortical organoids at day 50 of differentiation. Differential gene expression analysis of RNAseq of AD organoids compared to isogenic controls indicated activation of the ferroptotic pathway. This was also supported by results from untargeted proteomic analysis revealing significant changes between AD and isogenic brain organoids. Determining the causality between the development of Aβ plaques and the deregulation of molecular pathways involved in ferroptosis is crucial for developing potential therapeutic interventions.

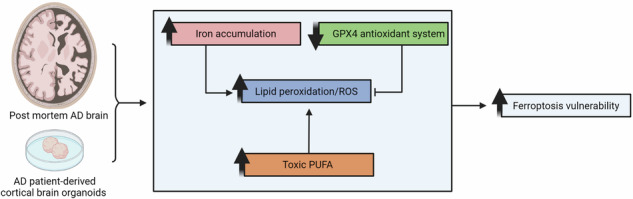

## Introduction

Alzheimer’s disease (AD), the most common form of dementia, affects over 44 million people worldwide and its prevalence is constantly increasing with the aging population [1]. Many treatment strategies aiming to directly disrupt the formation of amyloid β (Aβ) plaques and neurofibrillary tangles (NFTs), the two major AD hallmarks, showed modest or no benefit [2]. The pathological mechanisms linking proteopathology and neuronal death have not yet been well characterised.

Several cell death pathways, including apoptosis [3], necrosis [4], and autophagy [5] have been described in AD brain [6–8]. More recent findings suggest that ferroptosis, the iron accumulation-related and lipid peroxidation-driven type cell death pathway [9, 10], plays an important role in AD pathology [11]. Iron accumulation [12] and lipid peroxidation [13, 14], two ferroptosis hallmarks, have been detected during early AD pathology. In addition, mouse and human transcriptomic studies show that many ferroptosis-related genes are differentially expressed in AD pathology [15]. The gene expression of *Gpx4*, one of the most important antioxidant enzymes and the key ferroptosis regulator, is increased in the brain of AD mouse model compared to WT [16]. In the human AD brain *GPX4* is downregulated at an early stage of the pathology, while upregulated at a later stage of the pathology [17]. A comparable pattern of downregulation at early stages and upregulation at later stages of AD pathology was observed with many other ferroptosis-related genes expressed in excitatory/inhibitory neurons, astrocytes and oligodendrocytes [15, 17]. These findings together with observed difference in microglia between the expression of ferroptosis-related genes in Aβ-related versus tau-related pathology [18] suggests that vulnerability to ferroptosis might be related to both increased Aβ aggregation and tau hyperphosphorylation. However, whether ferroptotic cell death is triggered by Aβ aggregation, or tau hyperphosphorylation or whether there is an interaction between them remains yet to be confirmed.

The aim of this study was to investigate how Aβ pathology correlates with ferroptosis in human post-mortem brain, and whether ferroptosis inhibition could have a protective effect on Aβ-related pathophysiology. Our results reveal a deregulation of the expression of ferroptosis-related markers in AD post-mortem brain. In addition, we show that inhibition of ferroptosis can result in attenuation of Aβ-related lipid peroxidation and an increase in the expression of iron storage proteins in brain cortical organoids differentiated from iPSCs of AD patients compared to their isogenic controls. Differential expression analysis of AD organoids compared to isogenic controls indicated activation of the ferroptotic pathway. Altogether, our findings support the implication of ferroptosis in AD and underline the potential role of anti-ferroptotic drugs to prevent neurodegeneration and slow down the progression of AD.

## Materials and methods

### Human brain tissue and neuropathology

The use of post-mortem brain tissues from the BRAIN cohort (Brain Research in Aging, Inflammation and Neurodegeneration) was evaluated by the METc (nr 2021/496) on August 31, 2021, concluding that the protocol is not clinical research with humans as meant in the Medical Research Involving Human Subjects Act. The LTc UMCG (local review board) positively reviewed the described protocol (research registration nr 202100608). Formalin-fixed paraffin-embedded (FFPE) brain tissue from the right occipital cortex (OC) (at the level of Brodmann area 17 (BA17), area striata) of the neocortex was obtained from twelve subjects. We selected BA17 as the key brain region of interest to distinguish between AD patients with Aβ only and those with both Aβ aggregates and Tau hyperphosphorylation. This choice is informed by the fact that, in AD, the OC is initially affected by Aβ, whereas disease progression leads to subsequent Tau hyperphosphorylation in this brain area. Subjects were divided into four groups based on the presence or absence and progression of pathology: (a) controls (3 subjects; no Tau no Aβ plaque), (b) AD1 (2 subjects; no Tau in Brodmann area 17, 18 and Aβ plaque density moderate: 1/2 and plaque high: 1/2), (c) AD2 (4 subjects; Braak Tau stage 5 and plaque moderate:1/4 and plaque high: 3/4) and (d) AD3 (3 subjects; Braak Tau stage 6 and plaque high: 3/3). AD patients were neuropathologically diagnosed according to the commonly used criteria [19] and [20]. While Aβ pathology was on average constant among the different AD groups, the progression of tau pathology increased from no (depicted as AD1) to high (depicted as AD3).

### Immunohistochemistry and imaging of human slices

The 3-µm-thick sections were cut from paraffin blocks of donors and fixed on adhesive Starfrost glass slides. After de-paraffinization and rehydration, sections underwent a heat-induced epitope retrieval by microwave heating in 0.1 M TRIS hydrochloride (HCl) (pH 9.0) for 15 min. H_2_O_2_ diluted in PBS (0.3%) was used to block the endogenous peroxidase for 30 min. After washing, sections were blocked with the Avidin/Biotin Blocking Kit (Vector Laboratories, SP-2001) if GARbio/RAGbio secondary antibodies were used (for ferroportin and GPX4). Sections were incubated with rabbit or goat primary polyclonal antibodies (Table 1), which were diluted in 1% bovine serum albumin (BSA)/PBS. The incubation was 60 min at room temperature (RT) unless stated otherwise (Table 1). After washing, sections were incubated 30 min with horseradish peroxidase-labelled goat-anti-rabbit (GARPO) or rabbit-anti-goat (RAGPO) secondary antibodies diluted in 1% BSA/PBS + 1% normal human (AB) serum to stop unspecific binding. For better results, sections were washed again and incubated with a third corresponding antibody in 1% BSA/PBS + 1% AB serum for another 30 min. For visualisation, the sections were stained with 3,3-diaminobenzidine (DAB) 0.03% H_2_O_2_ in PBS for 10 min and then washed with demi water. Counterstaining was performed with haematoxylin. Sections were dehydrated through rising concentrations of ethanol, mounting medium and coverslips. Hamamatsu Nanozoomer was used to image the sections. AD subjects at different stages of the pathology and healthy controls were included in our analysis. We stained serial sections with 6 antibodies alternating with stainings for Aβ and p-Tau routinely used for AD diagnosis, to be able to perform correlation analysis later on. This allowed us to investigate the link between Aβ pathology and ferroptosis in post-mortem brain.

**Table 1:** Antibodies used for immunohistochemical analysis of ferroptosis-related markers and AD pathology in alphabetical order.

Primary antibody					Secondary antibody	Tertiary antibody
Target	Dilution	Manufacturer	Origin	Incubation	(Targtet, dilution (manufacturer))	
Aβ (6F/3D)	1:400	DAKO, M087	Mouse	60 min, RT	I-view + Ampl	–
Cytochrome C	1:500	Abcam, ab90529	Rabbit	60 min, RT	GARPO, 1:100 (DAKO, P0448)	RAGPO, 1:100 (DAKO, P0449)
Ferritin	1:50	LSBio, B14992	Rabbit	60 min, RT	GARPO, 1:100 (DAKO, P0448)	RAGPO, 1:100 (DAKO, P0449)
Ferroportin	1:50	LSBio, B1836	Rabbit	ON 4 °C	GARbio, 1:300 (DAKO, E0432)	StrepPO, 1:300 (DAKO, P0397)
GPX4	1:100	Abcam, ab116703	Goat	ON 4 °C	RAGbio, 1:300 (South. Biotech. Ass, 6165-08)	StrepPO, 1:300 (DAKO, P0397)
NCOA4	1:50	Invitrigen, PA5-96398	Rabbit	60 min, RT	GARPO, 1:100 (DAKO, P0448)	RAGPO, 1:100 (DAKO, P0449)
AT-8/Tau	1:20	Invitrogen MN1020	Mouse	60 min, RT	Ultra IVIEW	–
4HNE	1:200	Bioss, bs-6313R	Rabbit	60 min, RT	GARPO, 1:100 (DAKO, P0448)	RAGPO, 1:100 (DAKO, P0449)

*AB* antibody, *Aβ* amyloid beta, *Ampl* amplification, *GARbio* goat-anti-rabbit biotin, *GARPO* goat-anti-rabbit peroxidase, *GPX4* glutathione peroxidase 4, *min* minute, *NCOA4* nuclear receptor co-activator 4, *ON* overnight, *RAGbio* rabbit-anti-goat biotin, *RAGPO* rabbit-anti-goat peroxidase, *RT* room temperature of ~21 °C, *StrepPO* streptavidin peroxidase, *4HNE* 4-hydroxy-2-nonenal.

Microwave treatment of three times 10 min in Tris/HCl buffer (pH 9) was used as the antigen retrieval method for all antibodies against ferroptosis-related markers. Three-minute treatment with 98% formic acid at RT was used as antigen retrieval method for Aβ antibody and no antigen retrieval method was used for tau. The diagnostic stainings, AT-8/Tau and Aβ (6F/3D), were stained on the Benchmark (Vetana) which did not require a tertiary antibody step.

### Quantitative analysis of IHC human post-mortem brain sections

Scanned human brain sections were analysed by ImageScope where the image was split into four separate colour channels (blue: negative, yellow: weakly positive, orange: moderately positive and red: strongly positive pixels). To quantify the distribution and cellular localisation of the ferroptotic-related markers, we used positive pixel density scoring as reported previously [21, 22]. For the comparison between white and grey matter, three randomly encircled areas of comparable size both in white matter and grey matter were analysed and averaged per subject. For the comparison between amyloid beta and non-amyloid beta-containing areas, four plaque-containing areas and four adjacent non-plaque-containing areas of comparable size were selected and analysed per subject. For cross-sections of 100-day-old organoids, four to five organoids per condition were included in the analysis and the entire organoid cross-section was selected for the analysis. The thresholds for Iwp (high), Iwp (low) and Ip (low) were 200, 140, 80 for 4HNE, 200, 125, 75 for Cytochrome c, 200, 125, 75 for ferritin, 200, 115, 70 for ferroportin, 200, 150, 70 for GPX4 and 200, 135, 70 for NCOA4, respectively. In all cases, the selected and analysed areas were normalised to the number of total pixels selected and the blue channel was used as the threshold to remove the background from hematoxylin staining and orange and red were used to calculate positive pixel density based on the following formula:

$$\frac{\left({number\; of\; red\; pixels}\times 2\right)+({number\; of\; orange\; pixels}\times 1)}{{total\; number\; of\; pixels}}$$

### Iron measurement of human brain tissue

Brain tissue was obtained from the NeuroBiobank of the Institute Born-Bunge (NBB-IBB), Wilrijk (Antwerp), Belgium (ID: BB190113), and donors gave informed consent to donate their brains to the NBB-IBB. The medical ethics committee of the Hospital Network Antwerp (ZNA, approval numbers 2805 and 2806) granted the ethical approval and the study was compliant with the World Medical Association Declaration of Helsinki on Ethical Principles for Medical Research Involving Human Subjects. Human tissue collected as previously described were drained, and the wet weight was recorded. After lyophilization, samples were digested with nitric acid (HNO_3_, 65% Suprapur, Merck) for overnight digestion. Digestion was completed by heating the samples to 90 °C for 20 min, followed by dilution into an equal volume of hydrogen peroxide (30% Aristar BDH) and incubation for ~30 min period, allowing for effervescence to stop. Samples were then heated for 15 min at 70 °C. The average reduced volume was recorded, and the samples were further diluted with 1% HNO_3_ in water. Measurements were made using an Agilent 7700 series ICP-MS instrument under routine multi-element operating conditions using a Helium Reaction Gas Cell. The instrument was calibrated using 0, 5, 10, 50, 100 and 500 ppb of certified multi-element ICP-MS standard calibration solutions (ICP-MS-CAL2-1, ICP-MS-CAL-3 and ICP-MS-CAL-4, Accustandard) for a range of elements. A certified internal standard solution containing 200 ppb of Yttrium (Y89) was used as an internal control (ICP-MS-IS-MIX1-1, Accustandard).

### Differential expression of ferroptosis-related genes in Alzheimer’s disease

We used a publically available RNAseq dataset that compared the gene expression of AD patients at different stages of the pathology [23], and we analysed which of the 51 ferroptosis-related genes are differentially expressed at different stages of AD (Supplementary Table 1). In this dataset, ten male participants with an APOE ε3/ε3 genotype were selected (aged between 50 and 91) and ~6000 nuclei from the superior frontal gyrus (SFG) per individual were analysed. Results were reported separately for each cell type, including: neurons (excitatory and inhibitory) and glia cells (astrocytes, oligodendrocytes and microglia). Cytoarchitectonic criteria and histopathological validation were used to sample brain regions for snRNA-seq. Beta regression [24] was performed using the betareg package (version 3.1-1) in order to determine statistical significance.

### Chemical compounds

Human recombinant Amyloid β Protein Fragment 1-42 (Aβ1-42) was purchased from rPeptide, USA (Cat. No: A-1002-1); ferrostatin-1 (depicted in figures as Fer-1) was purchased from Sigma-Aldrich, USA (Cat. No. 347174-05-4). Stock solutions were prepared and dissolved following manufacturers instructions. Corresponding volumes of the stock solutions were diluted in culture medium to reach the final concentrations listed, and culture medium was used as a vehicle.

### Cell culture

Human induced pluripotent stem cells (iPSCs:AD4 harbouring the PSEN1-ΔE9 mutation, isogenic control corrected for the PSEN1-ΔE9 mutation and healthy control stem cell lines were kindly provided by Dr. Šárka Lehtonen and Prof. Jari Koistinaho [25] and differentiated to neuron progenitor cells (NPCs) according to the previously published protocol [7, 26].

### Proteomic analysis of neuron progenitor cells from AD patients and isogenic controls

Neuron progenitor cells were derived from human iPSCs (AD4 harbouring PSEN1-ΔE9 mutation, isogenic control corrected for PSEN1-ΔE9 mutation, and healthy control stem cell lines). Protein pellets were dried and then resuspended in either 47.5 µl (control) or 87.5 µl (AD) of 50% trifluoroethanol (TFE) in 50 mM ammonium bicarbonate (ABC). In total, 10 µl of each sample was taken for the protein estimation (BCA assay, Thermo). To the rest of the sample, 2.5 µl of 100 mM of the tris (2-carboxyethyl) phosphine (TCEP) in 50 mM ABC (pH = 7.5) was added, followed by 30 min incubation at 37 °C with shaking. After the reduction, a second alkylation step was carried out by adding 5 µl of 100 mM d5-NEM. Samples were then left for 20 min on RT, after which they were diluted 1 + 4 with ammonium bicarbonate (25 mM, pH = 8.5) and then digested overnight with trypsin (1 µg of trypsin per sample). On the following day, 3–4 µg of each sample was offline desalted using in-house made SDB-RPS stage tips (Empore, Supelco). Samples were dried down upon desalting, then resuspended in 12 µl of running buffer A. In all, 2 µl of each sample was used for liquid chromatography-tandem mass spectrometry (LC-MS/MS) based proteomics analysis.

Peptides were separated on an Ultimate 3000 RCS Nano Dionex system equipped with an Ionopticks Aurora Series UHPLC C18 column (250 mm × 75 µm, 1.6 µm) (Ionopticks). Total LC-MS/MS run per each fraction/run was 133 min with the following gradient (solvent A is 0.1% formic acid in water; solvent B is acetonitrile containing 0.1% formic acid): 0–18 min: 2% B; 18–100 min: 2–25% B; 100–107 min: 25–35% B, 107–108 min: 35–95% B, 108–118 min: 95% B; 118–118.1 min: 95–2% B; and 118.1–133 min: 2% B at a flow rate of 300 nL/min and 50 °C. The maXis II ETD mass spectrometer (Bruker) was operated with the captive source in positive mode employing the following settings: mass range: 200–2000 *m/z*, 2 Hz, capillary 1600 V, dry gas flow 3 L/min at 150 °C, nanoBooster 0.2 bar, precursor acquisition control set to fragment the top 20 most abundant peaks. Consequnt label-free protein quantification (LFQ), was performed using MaxQuant (v1.6.1.0; [27, 28]. False discovery rate (FDR) for database matching was set to 1% and minimum peptide length to six amino acids. Match between run feature was enabled with the match and alignment windows of 1 and 20 min, respectively. NEM and d5-NEM cysteine modifications were selected as dynamic modifications, and no static modifications were defined. At least two peptides were required for quantification. In Perseus [29], the table with protein LFQ intensities was as well filtered for contaminants, after which the matrix was further filtered to contain at least three valid values in at least one of the groups. Missing values were imputed from a normal distribution (width 0.3, downshift 1.8) and two-sample *t* tests corrected for multitesting were performed between the groups (FDR 1%, S0 (similar to log fold change) 2).

### Differentiation of organoids

The cBOs were generated from the above-mentioned iPSCs (AD4 harbouring PSEN1-ΔE9 mutation, isogenic control corrected for PSEN1-ΔE9 mutation and healthy control stem cell lines) (Supplementary Fig. 1). iPSCs were maintained on 6-well plates coated with Matrigel (Corning, USA #356234) in Essential 8^TM^ flex medium (E8F; Gibco^TM^, Thermo Fisher Scientific, Netherlands # A2858501) at 37 °C, 5% CO_2_. At 80–90% confluency, a day before spheroid formation, cells were pretreated with 1% dimethyl sulfoxide (DMSO; Merck Life Science N.V, Sigma, Netherlands #S002D) to improve spheroid differentiation capacity [30]. Cells were first washed with phosphate buffer saline (PBS; Gibco, Thermo Fisher Scientific, Netherlands #70011044) and detached with Accutase® (Stemcell Technologies, USA #07920) for 5 min at 37 °C. The dissociated single cells were seeded on AggreWell^TM^800 plates (Stemcell Technologies, USA #34850) containing 300 microwells per well, pretreated with AggreWell™ rinsing solution (Stemcell Technologies, USA #07010), according to the user’s manual. Cells were spun down at 100 rcf for 3 min and each single microwell contained at least 5000 cells, cultured in E8F medium (without antibiotic) containing 10 μM ROCK inhibitor Y-27632 (10 mM stock, Stemcell Technologies, USA #72304). After 24 h of incubation, spheroids were collected from the microwells using cut 1000 μL pipette tip and transferred into a sterile 10-cm Petri dishes in cortical differentiation medium [E6F with 10 μM dorsomorphin (DM; 25 mM stock, Stemcell Technologies, USA #72102), 10 μM SB-431542 (10 mM stock, Stemcell Technologies, USA #72234) and 10 μM XAV-939 (10 mM stock, Stemcell Technologies, USA #72674)]. The medium was changed every day for 6 days. At day 6, medium was changed into neuronal maintenance medium consisting of: Neurobasal (Gibco^TM^, Thermo Fisher Scientific, Netherlands #21103049) containing 1:50 B-27 supplement without vitamin A (Gibco^TM^, Thermo Fisher Scientific, Netherlands #12587010) and 1:100 GlutaMax (Gibco^TM^, Thermo Fisher Scientific, Netherlands #35050061). This medium was supplemented with 20 ng/mL epidermal growth factor (EGF; 100 μg/mL stock, Stemcell Technologies, USA #780061) and 20 ng/mL fibroblast growth factor 2 (FGF2; 100 μg/mL stock, Stemcell Technologies, USA #781341) from day 6 to day 16 with daily medium change. From day 16 to day 24, medium was changed every other day with fresh EGF and FGF2 added every time. At day 24, neuronal maintenance medium was supplemented with 20 ng/mL brain-derived neurotrophic factor (BDNF; 100 μg/mL stock, Stemcell Technologies, USA #78133) and 20 ng/mL Neurotrophin-3 (NT3; 100 μg/mL stock, Stemcell Technologies, USA #78074) until day 43, with media change every other day. cBOs were maintained in neuronal maintenance medium without growth factors from day 43 with media change every other day. cBOs used in this study were between 50 and 100 days old and treatments with either ferrostatin-1 or BACE inhibitor (BACE inh) started between day 34 and 37, and continued until day of collection (day 50 or 100). The inhibitors were replenished at every media change until collection.

### Immunofluorescent staining in iPSC-derived brain organoids

Day 50 and day 100 of cBOs of different conditions embedded in OCT were sectioned at 10 µm using the cryotome (Microm HM525, Thermo Fisher Scientific). Antigen retrieval with 10 mM sodium citrate buffer (Merck Life Science N.V, Sigma, Netherlands #6132043) was done on OCT sections in a pressure cooker for 30 min. Slices were then permeabilized with 0.1% Tween in PBS for 15 min and subsequently blocked with 5% normal goat serum (NGS; Merck Life Science N.V, Sigma, Netherlands, #NS02L-1ML) in PBS for 30 min. Overnight incubation at 4 °C was done with mouse anti-human Amyloid β (1:100, FUJIFILM Wako Pure Chemical Corporation 017-26871, BAN50) in blocking solution (5% normal goat serum in 0.1% Tween PBS). After primary antibody incubation, slices were then washed with PBS three times and incubated with secondary antibody donkey anti-mouse IgG (H + L) Alexa Fluor™ 488 (1:1000, Thermofisher Scientific, A-21202) in blocking solution for 90 min. OCT sections were then mounted with Fluoroshield™ with DAPI (4′,6-diamidino-2-phenylindole; Merck Life Science N.V, Sigma, Netherlands #F6057) prior to imaging. Immunofluorescent images were taken on a fluorescence Nikon microscope.

For immunohistochemical staining with 4HNE and ferritin, the same antibodies and protocol were used as for human brain sections. Protein expression was quantified by using QuPath-0.4.3 analysis tool.

### RNA isolation

Brain organoids were collected on Day 50 of the protocol. Isolation of RNA was performed using the Nucleospin® RNA isolation Kit (Macherey-Nagel). The samples were stored at −80 °C until they were sent for transcriptomic analysis.

### RNAseq library preparation, quality control and analysis

The quality of the samples, the construction and the sequencing of the RNA-libraries were performed by GenomeScan Bv. A set of standard quality metrics for the raw dataset was determined using third-party (FastQC v0.11.9) and in-house (FastQA v 3.1.25) QC tools. Human raw reads were aligned to the GRCh38.p13 genome. The reads were mapped to the reference sequence using a short read aligner based on Burrows-Wheeler transform (Tophat v2-2.1) with default settings. Reads were counted with HTSeq (v0.11.0). Only unique reads that fell within exon regions were counted. Raw count matrices were loaded in R. Lowly expressed genes were filtered using a cutoff of 0.5 CPMs. Only genes with >0.5 CPMs in at least three samples were included in the analysis. The count matrix was normalised with the blinded variance-stabilising method from DESeq2 with subsequent differential gene expression analysis 74. Normalised counts of organoid samples were filtered for the 51 ferroptosis-related genes (Supplementary Table 1) and a hierarchically clustered heatmap was generated depicting gene z-score. Visualisations were made with the package ‘pheatmap’.

### Proteomic analysis of iPSC-derived organoids

Organoids were disrupted in 50 µL of lysis buffer (20 mM Tris, 150 mM NaCl, 1 mM EDTA, 1 mM EGTA, 1% Triton X-100, 20 mM β-glycerol phosphate, 1 mM sodium orthovanadate, 5 mM sodium floride) and sonicated for (20–30 s). In-gel digestion of 20 µg of the protein lysates was performed for the LC-MS-based proteomics analyses, as described previously [31]. The generated LC-MS raw data were processed with Spectronaut (version 18.5.231110) (Biognosys) using the standard settings of the directDIA workflow, except that quantification was performed on MS1, with a human SwissProt database (www.uniprot.org, 20422 entries). For the quantification, the *Q*-value filtering was set to the classic setting, with Local Normalisation without imputing. For downstream processing, Z-scores of proteins that had no missing values were calculated and depicted in hierarchically clustered heatmaps with the package ‘pheatmap’. The mass spectrometry proteomics data have been deposited to the ProteomeXchange Consortium via the PRIDE [32] partner repository with the dataset identifier PXD047941 with project name: The link between Amyloid β and ferroptosis pathway in Alzheimer’s disease.

### Statistical analysis

The ordinary one-way ANOVA was used to analyse the difference between white (WM) and grey matter (GM), and between disease groups. A two-tailed paired t test was used to determine the statistical significance of the differences between the area containing Aβ plaques and the area around it. Normal distribution was tested, and data was analysed using one-way analysis of variance (ANOVA) multiple comparisons or by the Kruskal–Wallis non-parametric test. Statistical significance was defined as *p < 0.05. All statistical analyses were performed with GraphPad Prism 8.4.2 (GraphPad Software, USA) for Windows.

## Results

### Influence of Aβ pathology on ferroptosis-related protein levels

**Fig. 1:**
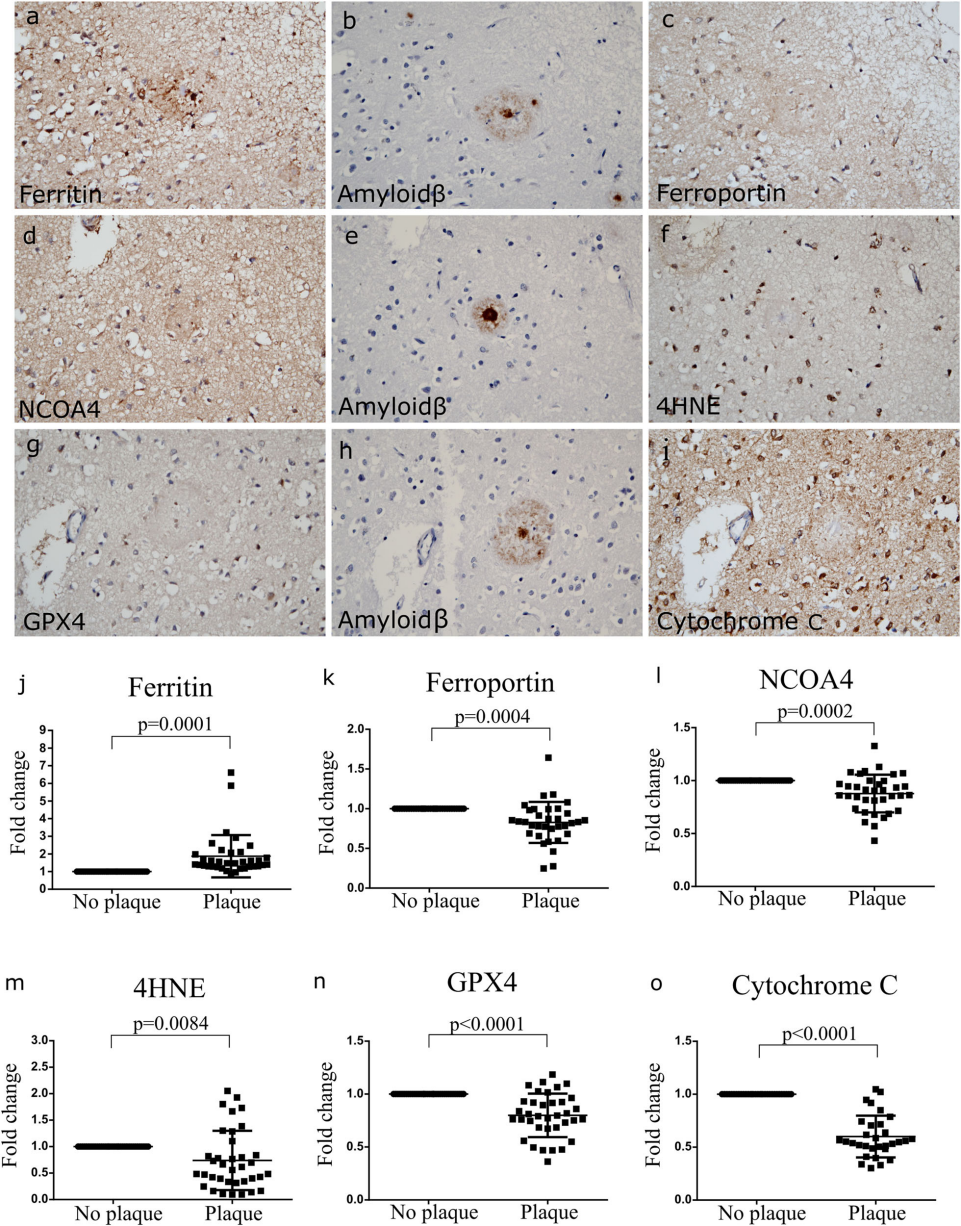
Ferroptosis-related markers in Aβ plaque and no-plaque-containing area in AD post-mortem brain. Example images of ferroptosis-related markers (**a**–**i**) and pixel densities outcomes (**j**–**o**) in relation to Aβ plaques in in OC (BA17). Each dot on the graph represents one plaque normalised to area next to it. Analysis included subjects from all three AD groups (AD1, AD2 and AD3). The pixel density of ferritin was higher in plaque compared to the area next to plaque (*p* = 0.001) (**j**) while ferroportin (*p* = 0.004) (**k**), NCOA4 (*p* = 0.002) (**l**), 4HNE (*p* = 0.0084) (**m**), GPX4 (*p* < 0.001) (**n**), and cytochrome C (*p* < 0.0001) (**o**) pixel density was lower in plaque compared to the non-Aβ containing area in the immediate vicinity. Results are normalised to no-plaque-containing area and represented as fold change. Two-tailed paired *t* test was used to determine the statistical significance between groups (*p* *<* 0.05).

To investigate the link between Aβ pathology and ferroptosis-related proteins in post-mortem AD brain tissue, we selected mature Aβ plaques in patients from several AD groups (termed AD1, AD2 and AD3 based on the pathology status, as described in Material and Methods section) and compared the expression of ferroptosis-related proteins in the Aβ plaque area with the adjacent area without any plaques (Fig. 1a–i). The mature plaques were selected based on the presence of neuroinflammation/gliosis, distorted neurites, and the presence of a dense core. Ferroptosis-related proteins linked to iron homoeostasis, oxidative stress, and lipid peroxidation were selected for the analysis. The measurements of the expression of ferroptosis-related proteins (ferritin, ferroportin, NCAO4, GPX4 and 4HNE) revealed significant differences between areas with and those without Aβ plaques (Fig. 1j–o). Positive staining for ferritin was observed in glial cells and distorted neurites (axons and dendrites). Levels of ferritin were significantly higher in the plaque compared to the adjacent areas (Fig. 1j), similar to what has been reported in the whole AD brain [33]. Ferroportin was detected as a weak cytoplasmic staining of glial cells and in neurites. The overall quantification of the ferroportin protein levels showed a significantly decreased expression in the Aβ plaque compared to the adjacent area (Fig. 1k). NCOA4, was detected in neurites, however the signal was significantly decreased in the plaque compared to the adjacent areas (Fig. 1l). Quantification of 4HNE and GPX4 signal in AD brain tissue showed that their expression was higher in the adjacent areas of Aβ plaques than in the plaque (Fig. 1m, n). Finally, cytochrome C expression was lower in the plaque compared to the adjacent area of the plaque (Fig. 1o). These results suggest that levels of the analysed ferroptosis-related proteins differ between inside and outside of the Aβ plaque.

### Differences between the expression of ferroptosis-related proteins in white and grey matter: a quantitative analysis

**Fig. 2:**
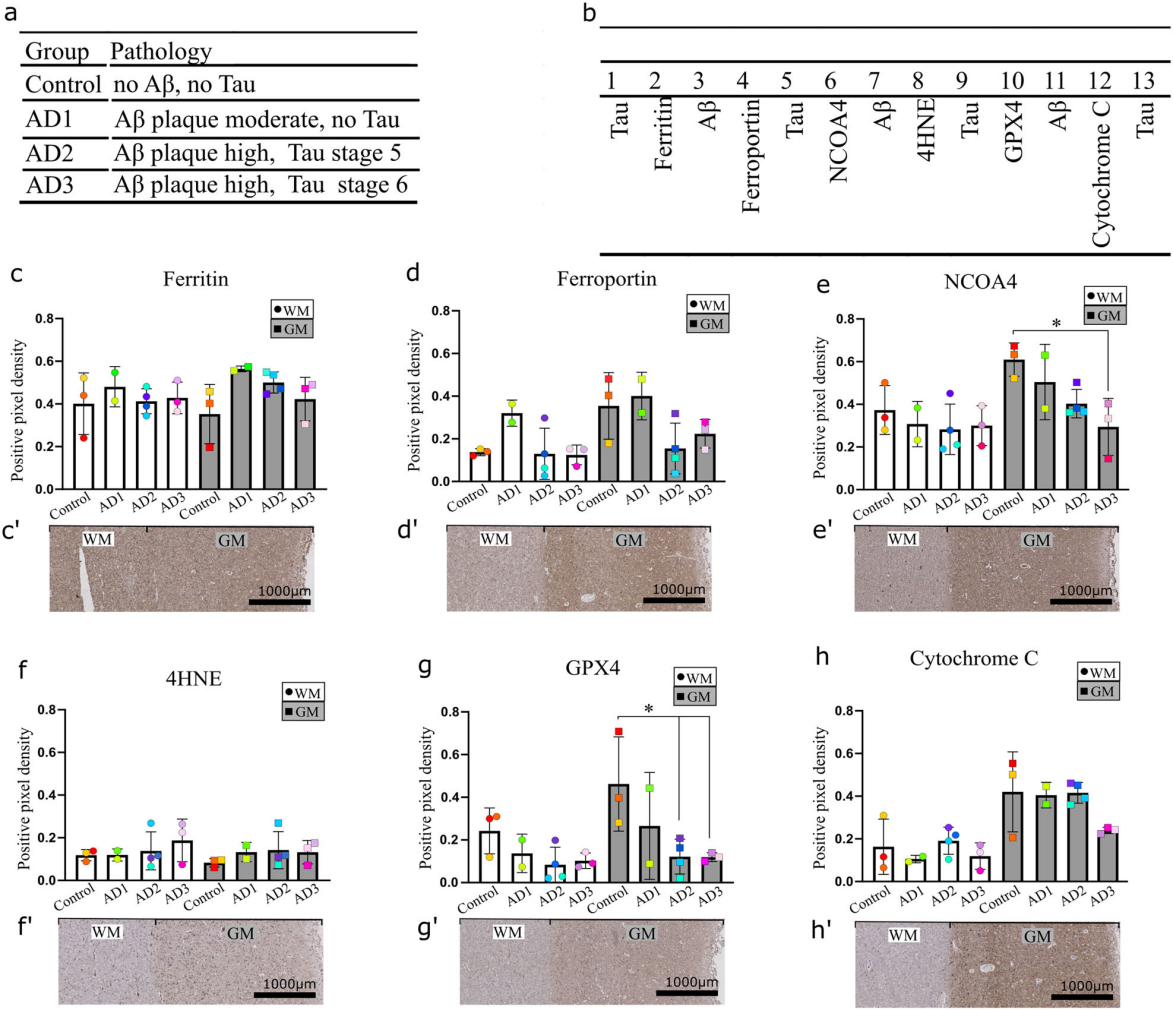
Multipanel figure depicting the levels of ferroptosis-related proteins at different stages of AD pathology. **a** Investigated groups, **b** order of stainings in OC (BA17) and quantification and example images of ferroptosis-related proteins in WM and GM in control and diseased subjects (**c**–**h**). No changes in ferritin protein (**c**) and 4-HNE (**f**) in any of the groups were observed. Higher expression of ferroportin (**d**), NCOA4 (**e**), GPX4 (**g**) and cytochrome c (**h**) was observed in GM compared to WM. Both NCOA4 and GPX4 protein expression in GM significantly decreases with increasing severity of AD pathology (NCOA4 control vs AD3, *p* = 0.0424; GPX4 control vs AD2, *p* = 0.0352 and GPX4 control vs AD3, *p* = 0.0476). In WM, a similar trend can be observed although not significant. Each dot on the graph stands for the average of three annotated areas per subject. One colour per subject is consistent across all graphs. Ordinary one-way ANOVA was used to determine significance between groups (*p* < 0.05).

To examine whether there are alterations in the expression of protein markers related to ferroptosis between grey (GM) and white matter (WM), we analysed the ferroptosis-related proteins in individuals diagnosed with AD and healthy subjects (Fig. 2). Detailed description of the expression patterns and example images can be found in Supplementary Figs. 2 and 3. Following the description of the expression pattern of the ferroptosis-related proteins, we quantified their expression by pixel density scoring (Fig. 2a, b). Our analysis of the difference between groups in WM and GM (Fig. 2c–h) revealed that both ferritin and ferroportin showed no significant differences between healthy or disease groups. In GM, ferritin and ferroportin expression decreased in the later stages of AD disease severity. NCOA4 showed no significant change in the WM of AD patients independent of disease severity, while a gradual decrease of NCOA4 expression during the progression of the pathology was observed. The decrease of NCOA4 expression was significant in the late stage of AD pathological severity compared to control (Fig. 2e). GPX4 expression in WM seemed to decrease during the AD pathology, although not at significant levels (Control vs AD2 *p* = 0.2208). Similarly to NCOA4, GPX4 was significantly decreased in the late stage AD pathology (AD2 and AD3 groups) compared to control in GM. No changes were observed in 4HNE and cytochrome c stainings between the control and AD groups.

Together, these results indicate that the expression of ferroptosis-related proteins varies between WM and GM, and that NCOA4 and GPX4 expression decrease along with the increasing severity of the disease in GM. These results indicate that differentiating between white and grey matter, could allow for a more precise understanding of the disease’s impact on different brain regions.

**Fig. 3:**
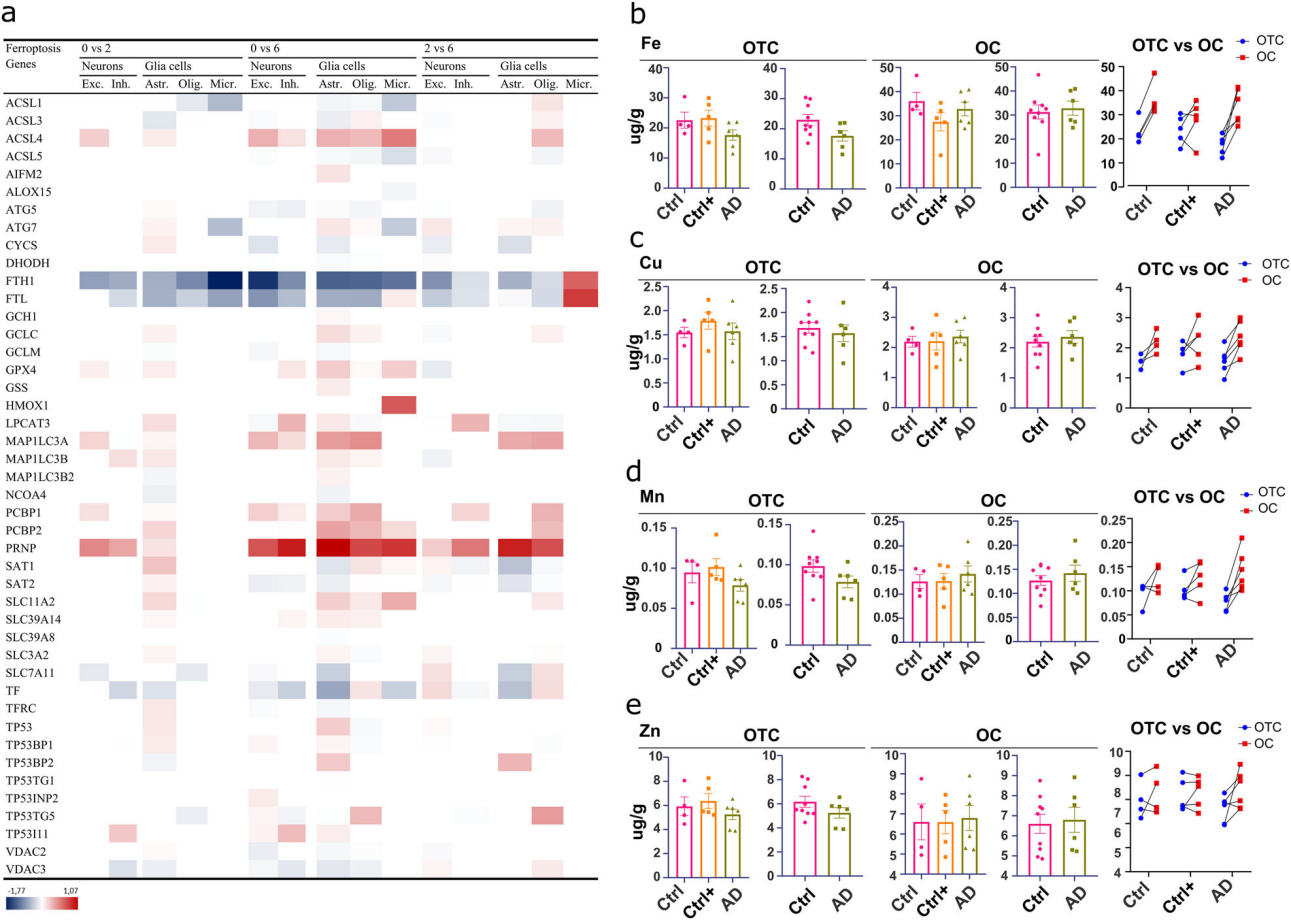
Ferroptosis-related gene expression and iron content in AD compared to healthy control human brain. **a** Average log2 fold change of ferroptosis-related DEGs in various cell types related to different Braak stages of AD. Decreased (blue) and increased (red) expression of ferroptosis-related genes in neurons (Excitatory (Exc) and inhibitory (Inh)) and glia cells (astrocytes (Astr), oligodendrocytes (Olig) and microglia (Micr)) in AD brain. White space corresponds to unchanged gene expression. Analysed brain area corresponds to the superior frontal gyrus (SFG) at the level of the anterior commissure (Brodmann area 8) of patients with different Braak stages (0 vs 2, 2 vs 6, and 0 vs 6). Early AD is associated with a decrease and late AD with increase in ferroptosis-related gene expression. The criteria to determine if the change in the gene was significant included the false discovery rate (FDR)-corrected *p* < 0.05 in a two-sided Wilcoxon rank-sum test, absolute log2 >0.25, and FDR-corrected *p* < 0.05 in a Poisson mixed model. Data was analysed based on [23]. **b** Levels of iron (Fe), **c** copper (Cu), **d** manganese (Mn) and **e** zinc (Zn) in AD and control patients. AD donors included those with amyloid-β pathology without tauopathy in the occipital cortex (OC) and occipitotemporal cortex (OTC), while control donors were either pathology-free (Ctrl) or had low amyloid-β levels without tau (Ctrl + ). Iron content in the occipital (OC) and occipitotemporal cortex (OTC) does not differ between AD and control patients. No statistical significance was detected between groups (Control (Ctrl), Control +versus (Ctrl + ) and Alzheimer’s disease (AD)).

It has been previously shown that differential expression of ferroptosis-related genes in AD affects various cell types, and it was suggested that the observed changes could be related to different stages of the pathology [15]. To follow up on this, we investigated whether ferroptosis-related genes are differentially expressed in different Braak stages of AD (Fig. 3a).

We screened publicly available single nucleus RNAseq (snRNA-seq) datasets generated by Leng et al. [23]. First, we investigated whether the expression of ferroptosis-related genes is affected by the severity of the AD pathology, determined by the Braak classification (Braak stages 0, 2 and 6) [23]. We observed that the ferroptosis-related genes are differentially expressed in all analysed cell types. Most of these ferroptosis-related genes seem to be differentially expressed at Braak stage 6 compared to Braak stage 2 (number of DEGs in 0 vs. 2 < 0 vs. 6). Furthermore, the fold change observed in neurons at Braak stage 2 seems to be increased with increased tau pathology. In excitatory neurons, a stronger decrease of *FTH1* was observed between Braak stage 0 vs. 6 compared to Braak stage 0 vs. 2. Similarly, a stronger increase of *ACL4* and *PRNP* was observed between Braak stage 0 vs. 6 compared to Braak stage 0 vs. 2. Finally, ferroptosis-related genes deregulation in glial cells seems to be associated with later stages of tau-associated AD pathology, as not only more DEGs could be observed with increased pathology, reflected by a higher Braak stage, but also more cell types were affected by AD pathology (number of DEGs in 0 vs. 2 < 0 vs. 6 and 0 vs. 2 < 2 vs. 6).

Next, we measured total iron (Fe) content alongside Copper (Cu), Manganese (Mn) and Zinc (Zn) levels in OC and OTC brain areas of AD patients and control individuals. In these brain areas, no significant changes in the iron content were observed between AD and control patients (Fig. 3b). Similar results were observed with Cu, Mn and Zn. In AD pathology, iron levels depend on the brain region [34], and it was reported being slightly increased in the AD hippocampus and cortex [12]. Although iron levels do not increase during ferroptotic cell death, an increase in cellular iron could increase the vulnerability of cells to cell death.

### Differential expression of ferroptosis-related genes and proteins in human AD iPSCs-derived brain organoids compared to isogenic brain organoids

**Fig. 4:**
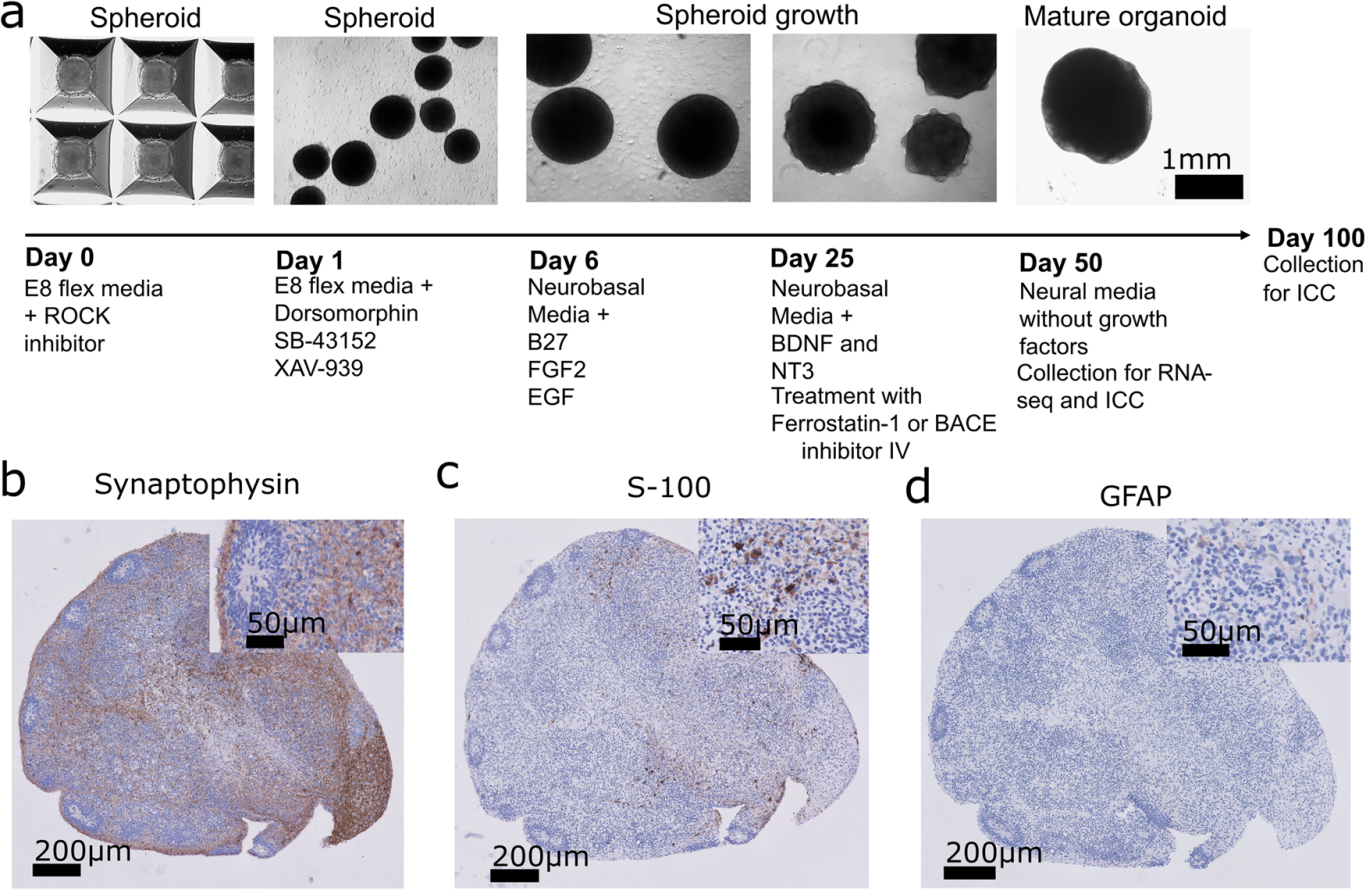
Development and characterisation of cerebral organoids at day 100. **a** Representative micrographs of the organoid generation protocol (Isogenic cortical brain organoids (Iso-cBOs) and AD-derived cortical brain organoids (AD-cBOs)) and **b** synaptophysin, **c** S-100 and **d** glial fibrillary acidic protein (GFAP) staining in FFPE Iso-cBOs at day 97. Briefly, iPSCs were seeded and aggregated into spheroids. After one day, spheroids were harvested and transferred into a non-adherent petri dish. Spheroids were differentiated accordingly until day 34, where they were treated with either ferrostatin-1 (Fer-1) or BACE inhibitor (to decrease Aβ aggregation). The inhibitors were replenished at every media change. The inhibitors were replenished at every media change. Organoids were matured until day 50 or day 100, when they were collected.

Our analysis of the post-mortem human AD brain showed that the expression of ferroptosis-related proteins is changed during AD pathology. However, post-mortem tissue provides only a static snapshot in time. To further explore how the ferroptotic pathway is affected by the progression of the pathology, we performed proteomic analysis on the neuronal progenitor cells (NPCs) differentiated from iPSC of PSEN1-Δ9 (depicted as AD-NPCs) and isogenic controls. Previously, it has been shown that iPSCs bearing this mutation express significantly lower GPX4 [35]. Here, we revealed significant decrease of Haem oxygenase (HMOX1) and Serotransferrin (TF) and significant enrichment of Glutathione synthetase (GSS) and ceruloplasmin (CP) in AD-NPCs compared to controls (Supplementary Fig. 1). These results suggest that at an early stage of iPSCs differentiation, AD-NPCs could have an increased resistance to ferroptosis. To better mimic the complexity of the human brain, we differentiated brain organoids from iPSC of PSEN1-Δ9 (depicted as AD-cBOs) and corresponding isogenic lines (depicted as Iso-cBOs) and studied whether ferroptosis-related proteins were altered in AD organoids. We generated human brain organoids and cultivated them for 100-day-old to allow maturation of neuronal cells (Fig. 4a), and the following stainings were performed to characterise the organoid composition: synaptophysin, S-100 and glial fibrillary acidic protein (GFAP) in the isogenic brain organoids (Fig. 4b–d). Throughout the manuscript, the analysis of these organoids was performed at 50 and 100 days of in vitro differentiation.

**Fig. 5:**
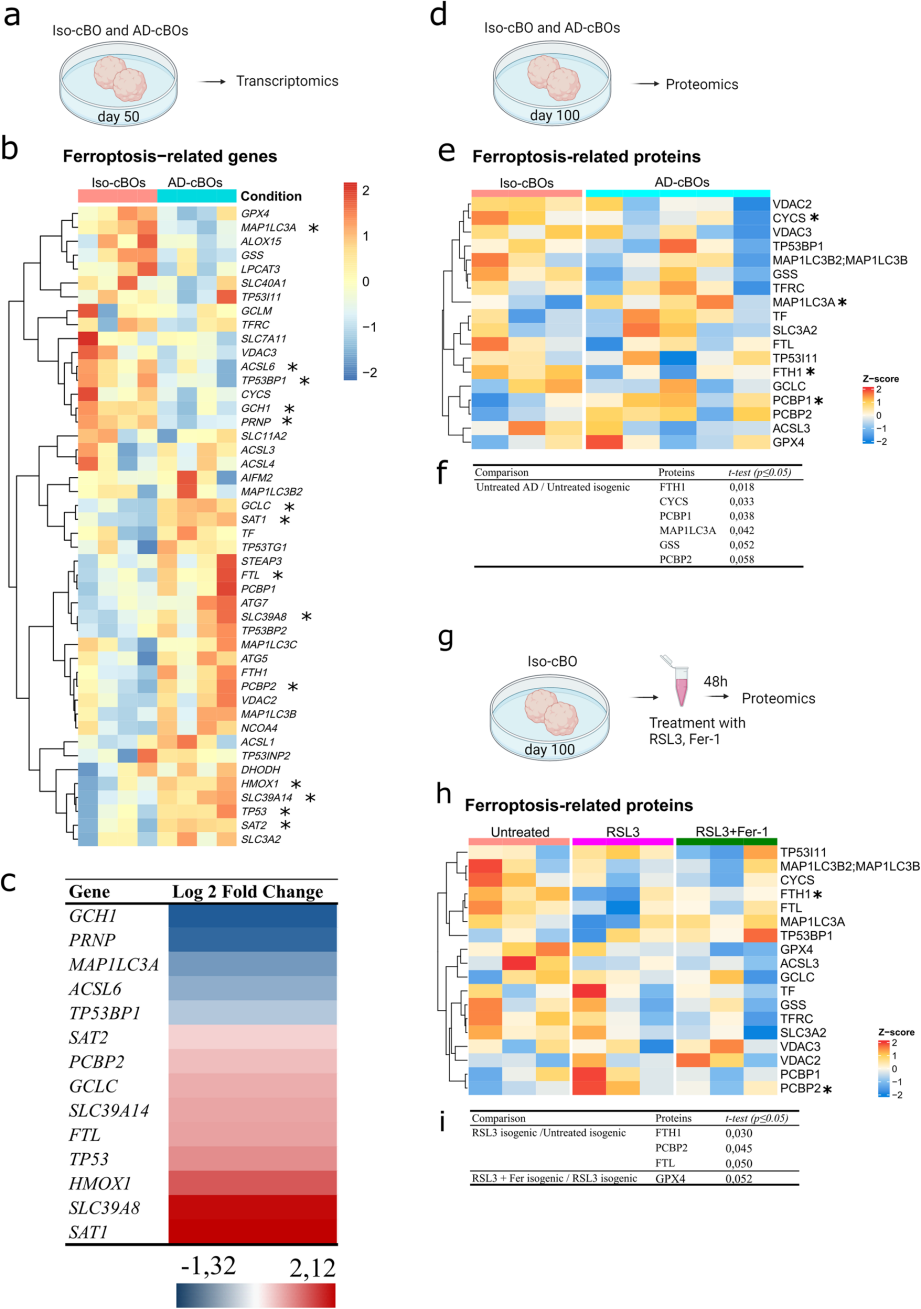
Difference in transcriptomic and proteomic ferroptotic signature between human AD-cBOs and their respective isogenic controls treated with or without ferroptosis inducer (RSL3) and/or inhibitor (Fer-1). **a** Schematic representation of an experimental setup (**b**) showing a hierarchically clustered heatmap depicting gene expression changes in ferroptosis-related genes in AD and Iso-cBOs as Z-scores. Visualisations were performed with the pheatmap package (*denotes *P*adj <0.05). **c** Heatmap depicting the Log2FoldChange of significant genes (*P*adj <0.05) of AD-cBOs compared to Iso-cBOs at day 50. DESEQ2 was used to determine the significant differences between the normalised counts of AD-cBOs compared to Iso-cBOs. **d** Schematic representation of an experimental setup (**e**) showing hierarchically clustered heatmap and **f** table depicting proteomic changes in ferroptosis-related proteins in Iso and AD-cBOs as Z-scores. Visualisations were performed with the pheatmap package cBOs (*denotes *p* value < 0.05 determined by *t* test). We identified 18 proteins exhibiting differential expression patterns within organoids derived from AD compared to control cBOs. **g** Schematic representation of an experimental setup (**h**) showing hierarchically clustered heatmap and **i** table depicting proteomic changes in ferroptosis-related proteins in untreated compared to treated (RSL3 and RSL3+Fer-1) Iso-cBOs as Z-scores. Visualisations were performed with the pheatmap package comparing RSL3-treated vs. untreated Iso-cBOs, and comparing RSL3-treated+Fer-1 vs RSL3 alone treated Iso-cBOs (*denotes *p* value < 0.05 determined by *t* test). For the quantification, the *Q*-value filtering was set to the classic setting, with Local Normalisation without imputing. For downstream processing, Z-scores of proteins that had no missing values were calculated and depicted in hierarchically clustered heatmaps with the package ‘pheatmap’. Raw data are available via ProteomeXchange with the identifier PXD047941. Part of the figure used BioRender programme.

We aimed to investigate the effect of the AD background on gene expression in the differentiated cerebral brain organoids. For this, we performed transcriptome analysis (Fig. 5a) of the AD- and- isogenic brain organoids and surveyed for the ferroptosis-related geneset [15] as depicted in Supplementary Table 1. When compared to their isogenic controls, AD organoids showed a downregulation of a cluster of genes which included *MAP1LC3A*, *ACSL6*, *GCH1*, *PRNP*, among others (Fig. 5b). Conversely, these organoids also exhibited upregulation of a cluster of genes which included *GCLC, SAT1, FTL, HMOX1, SLC39A14 TP53* and *SAT2* (Fig. 5b). Differential expression (DE) analysis between AD- and Iso-cBOs detected genes that were significantly different (Fig. 5c and Supplementary Table 2). These data provide evidence on the modulation of gene expression of ferroptosis-related genes in the AD organoid model.

Analysing protein levels provides stronger evidence about the actual translation of genes into proteins, surpassing the information obtained from gene expression analysis alone. Therefore, we also evaluted the expression changes of players involved in the ferroptosis pathway on protein level by untargeted proteomics (Fig. 5d). Filtering the proteomic dataset using the same list of ferroptosis-related markers as above, 18 proteins were differentially expressed in AD compared to isogenic brain organoids (Fig. 5e). AD-cBOs had significantly lower levels of cytochrome c (CYCS, *p* = 0.033) and ferritin heavy chain (FTH1, *p* = 0.018) and significantly higher expression of poly(rC) binding protein 1 (PCB1, *p* = 0.038) and microtubule-associated protein 1 light chain 3 alpha protein (MAP1LC3A, *p* = 0.042) compared to isogenic brain organoids (Fig. 5f). We next analysed the effects of modulating ferroptosis vulnerability in Iso-cBOs and treated them with a ferroptotic inducer, RSL3 and a ferroptotic inhibitor, Fer-1 (Fig. 5g). Incubation of isogenic brain organoids with RSL3 for 48 h resulted in a lowering trend of GPX4 protein levels compared to untreated isogenic brain organoids and significantly lower levels of FTH1 (*p* = 0.030) (Fig. 5h, i). On the other hand, the protein levels of poly(rC) binding protein 2 (PCBP2), the iron transporter protein involved in processes that include ferroportin-1 and herme oxygenase, were significantly higher in RSL3-treated compared to untreated isogenic brain organoids (*p* = 0.045) (Fig. 5h, i). These results suggest that inhibition of GPX4 could result in impairment of iron homoeostasis.

### Ferrostatin-1 prevents Aβ-like aggregates, reduces lipid peroxidation and boosts the expression of iron storage in AD-cBOs

**Fig. 6:**
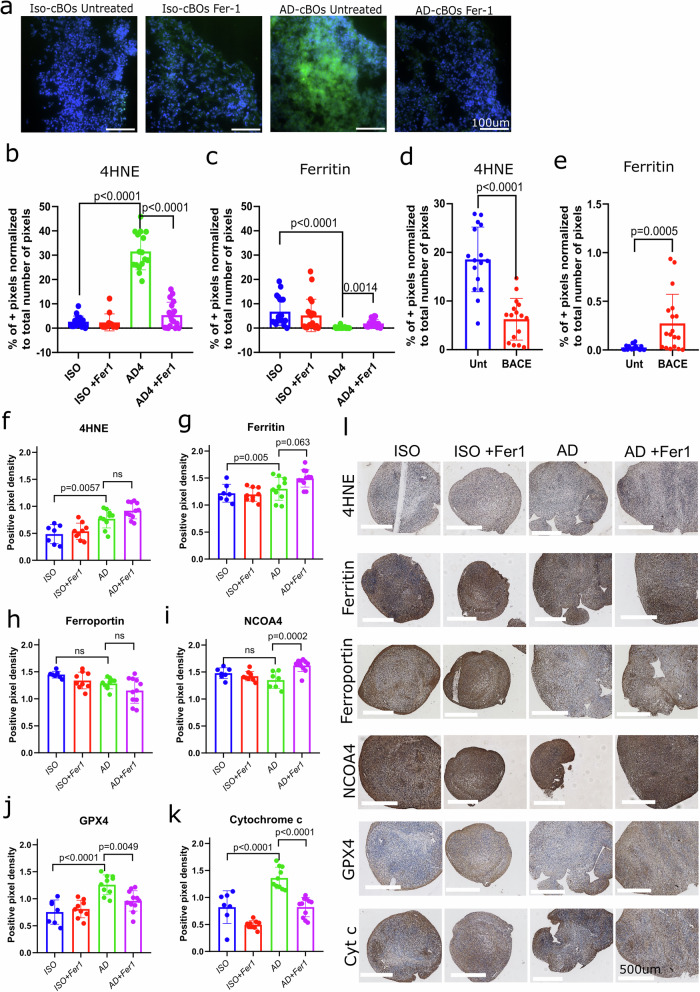
Ferroptosis inhibition prevents Aβ aggregate-like formation, reduces lipid peroxidation and boosts the expression of iron storage protein in human AD-cBOs at day 50 but not at day 100 of differentiation. Brain organoids were treated with either ferrostatin-1 (Fer-1) or BACE inhibitor (BACE) (to decrease Aβ aggregation) at day 34. The inhibitors were replenished at every media change. **a** Example images of organoids at day 50 stained with Aβ (green) which revealed Aβ-like aggregates in AD-cBOs while ferrostatin-1 (Fer-1) treatment two weeks prior to organoid harvesting reduced this Aβ aggregation. **b** Immunohistochemical analysis revealed higher level of 4HNE (*p* < 0.0001) and **c** lower level of ferritin (*p* = 0.0005) in AD-cBOs compared to Iso-cBOs at day 50. These changes were prevented by pretreatment with fer-1. **d** At day 100, BACE inhibition was associated with lower 4HNE (*p* < 0.0001) and **e** higher ferritn levels (*p* = 0.0005) in AD-cBOs. **f**–**k** Positive pixel density scoring of AD-cBOs compared to Iso-cBOs of various ferroptosis-related proteins and **l** representative images at day 100 are shown. In this analysis, we selected areas of the organoids that did not include signs of hypoxia and apoptosis. When excluding hypoxic cores from our analysis, we observed significantly higher levels of 4HNE (*p* = 0.005) (**f**), ferritin (*p* = 0.005) (**g**), GPX4 (*p* < 0.0001) (**j**) and cytochrome c (*p* < 0.0001) (**k**) in AD-cBOs compared to Iso-cBOs. Unlike at day 50, the increase in 4HNE and ferritin were not prevented by the pretreatment of fer-1 at day 100 (**f**, **g**). Fer-1 pretreatement decreased the levels of GPX4 (*p* = 0.0049) (**j**) and cytochrome c (*p* < 0.0001) (**k**) compared to the untreated AD-cBOs. No changes were observed in the levels of ferroportin (**h**), and NCOA4 levels were higher in AD-cBOs pretreated with fer-1 compared to the untreated AD-cBOs (*p* = 0.0002) (**i**). Unpaired *t* test was used to determine the significance of untreated compared to BACE inhibitor and ordinary one-way ANOVA was used to determine significance when more than two groups were compared.

Interestingly, Aβ-like aggregates were observed in 50-days-old AD brain organoids compared to isogenic brain organoids (Fig. 6a). Treatment with 1 μM Fer-1, a ferroptosis inhibitor, for 2 weeks, resulted in a reduction of extracellular Aβ deposition (Fig. 6a). At day 50, AD cerebral brain organoids showed higher 4HNE (Fig. 6b) and lower ferritin expression (Fig. 6c) compared to isogenic brain organoids. Both effects were reduced by ferrostatin-1 treatment (Fig. 6b, c). As a positive control, we used a β-Secretase inhibitor IV (BACE) to see if we can reduce the AD pathology. The BACE inhibitor is a cell-permeable, and potent inhibitor binding to BACE-1 and 2 active sites and blocking their proteolytic activity could result in reduced Aβ production [36]. BACE inhibitor treatment starting at 34 days of cBOs differentiation until 100-day-old cBOs, resulted in a significant reduction in 4HNE levels (Fig. 6d) and increased ferritin expression in AD compared to isogenic brain organoids (Fig. 6e). These results suggest that Aβ deposition can be correlated with the production of lipid peroxides and reduced capacity to store intracellular iron in AD brain organoids.

We further aimed to determine whether the observed correlation between Aβ pathology and heightened susceptibility to ferroptosis persist from day 50 to day 100 of cerebral brain organoids. To accomplish this, we conducted an analysis of protein levels using positive pixel density scoring of previously examined proteins in post-mortem human brains. At the day 100 of the differentiation of AD organoids, it’s essential to highlight that the pathology became advanced, potentially leading to severe cell damage due to increase in pathology and the presence of apoptotic bodies. Results from analysis without excluding hypoxic cores can be seen in Supplementary Fig. 4. We therefore excluded the hypoxic cores and analysed the changes in ferroptosis-related proteins at day 100 (Fig. 6f–k). The comparison of AD compared to Iso-cBOs revealed higher levels of lipid peroxidation (4HNE, *p*= 0.005) (Fig. 6f), iron storage (ferritin, *p* = 0.005) (Fig. 6g), GPX4 (*p* < 0.0001) (Fig. 6j) and cytochrome c (*p* < 0.0001) (Fig. 6k). However, the increased levels of 4HNE and ferritin in AD-cBOs were not prevented by the pretreatment of fer-1 at day 100 (Fig. 6f, g) as it was observed at day 50 (Fig. 6b, c). Fer-1 pretreatment decreased the levels of GPX4 (*p* = 0.0049) (Fig. 6j) and cytochrome c (*p* < 0.0001) (Fig. 6k) compared to the untreated AD-cBOs. No changes wetre observed in the levels of ferroportin (Fig. 6h), and NCOA4 levels were higher in AD-cBOs pretreated with fer-1 compared to the untreated AD-cBOs (*p* = 0.0002) (Fig. 6i).

In summary, at day 100 of organoid differentiation, our analysis revealed an advanced pathology, potentially leading to severe cell damage, despite Fer-1 treatment failing to prevent alterations in 4HNE expression levels observed between AD and isogenic brain organoids. Overall, the data from the human brain organoids demonstrate a clear alteration of ferroptotic-related genes and proteins, and a potential for inhibition of the ferroptotic pathway in the early phases of AD pathology.

## Discussion

In this study, we demonstrated that AD-related Aβ pathology is associated with changes in protein expression of ferroptosis-related proteins in the human brain. In addition, revealing that ferrostatin-1 treatment can mitigate Aβ-related effects on lipid peroxidation, and iron homoeostasis in AD iPSCs-derived brain organoids further supports the hypothesis that in AD, ferroptosis is linked with Aβ pathology. The summary of alterations in ferroptosis-related genes and proteins across various models used in this study can be viewed in Supplementary Table 3. Interestingly, the poly(rC)-binding protein (PCBP)1 and PCBP2 were found upregulated both at the gene (day 50, in culture) and protein (day 100, in culture) level in AD brain organoids. These multifunctional adaptor proteins function as iron chaperones and could deliver ferrous iron to ferritin, prolyl/asparagyl and deoxyhypusine hydroxylase. These hydrolyses act as mononuclear iron enzymes. Recently, PCBPs were shown to maintain nonheme iron cofactors in cytosolic enzymes, especially when iron becomes scarce [37]. A recent study showed that APP directly interacts with PCBPs, suggesting that PCBPs could be an important link between ferroptotic pathway and APP function [38]. Ferritin gene was found to increase in the human brain organoids (day 50) and also at the plaque site of the human AD brain. However, at later developmental stages of the brain organoids, ferritin was found decreased, as shown by proteomics. The expression of ferritin might fluctuate during the AD pathology, depending on its location and degree of pathology. A recent study showed that baseline CSF ferritin levels were negatively associated with cognitive performance over 7 years in MCI patients [39]. Our results improve the understanding of ferroptosis implications in AD and point to the potential of targeting ferroptosis, as a therapeutic strategy to prevent/slow down the development of AD pathology.

The results of comparing healthy controls and patients with Aβ pathology alone with Aβ together with tau pathology indicate that Aβ is associated with disturbances in iron balance, as evidenced by lower NCOA4 expression, and a decrease in GPX4 antioxidant enzyme expression in the grey matter. At later stages of AD, there is a noticeable reduction in NCOA4 levels in the grey matter, which supports the previously proposed hypothesis that long-term NCOA4 decrease makes cells more susceptible to ferroptosis [40]. While a temporary decrease in NCOA4 results in less breakdown of ferritin, leading to increased iron storage and therefore less available iron in the cell [41], long-term reduction in NCOA4 expression or function, as observed in animal studies, leads to an overload of iron and oxidative stress [42]. This suggests that a systemic decrease in NCOA4 can lead to an increase, rather than a decrease, in ferroptotic cell death. Furthermore, these findings align with the increased levels of iron and decreased autophagy observed in the brain during neurodegeneration [43]. To ensure that our findings are attributed to varying degrees of AD pathology and not external variables, we conducted correlation analyses between positive pixel density scoring and age, gender, post-mortem delay, and fixation time. No correlations were observed with any of these variables (Supplementary Fig. 5).

Regarding the pattern of NCOA4 expression in the GM, in a control healthy brain, NCOA4 was deposited near the plasma membrane in a punctuated manner. However, in AD cells, this pattern seems to be less evident. This suggests that NCOA4 in AD may be translocated to the cytoplasm, where it could induce the degradation of ferritin and increase sensitivity to ferroptosis [41]. However, it is unclear whether NCOA4 can bind to the cell membrane before translocating to the cytoplasm, apart from the evidence demonstrating its truncated isoform SRC3’s ability to bind to the cell membrane [44]. Another explanation for the decrease in punctuate NCOA4 staining is that while NCOA4 might be located in synapses in a control setting, and these synapses are lost in AD [45].

In our study, we observed decreased GPX4 protein expression in advanced stages of AD. Interestingly, previous research has shown upregulation of *GPX4* at the transcriptome level during these stages [15]. This phenomenon, similar to what has been proposed in Parkinson’s disease [46], suggests a compensatory mechanism where cells increase transcription to compensate for reduced GPX4 protein levels. These findings support the hypothesis that neurodegeneration in AD is linked to ferroptosis.

Our analysis of the association between ferroptosis and the key pathological hallmarks of AD revealed significant changes in ferroptosis-related proteins. Aβ pathology showed associations with increased iron storage, decreased iron export, reduced degradation of ferroportin, decreased GPX4 levels, and decreased mitochondrial content within Aβ plaques. Additionally, we observed increased lipid peroxidation in the vicinity of the plaque. These findings support previously published results showing that also on a genetic level, ferroptosis-related protein expression in the brain differs between patients who died early compared to later stages of AD pathology [15]. Furthermore, neural stem cells seem to be more resilient to different death stimuli [47–49]. Our post-mortem analysis revealed that proteins associated with ferroptosis exhibit varying expression levels in GM across brain tissue exhibiting different stages of pathology. Specifically, the observed changes between controls and individuals with AD were more pronounced at higher severities of AD pathology compared to the differences observed between controls and those with early-stage AD. However, whether the potential resistance of NPCs is an event associated with a compensatory mechanism of AD cells at early stages, or if it also occurs at later stages of differentiation remains to be elucidated.

In this research, we showed that ferrostatin-1 can prevent Aβ-like aggregation, restore ferritin levels, and decrease lipid peroxidation in brain organoids derived from human AD iPSCs. This supports the hypothesis that Aβ can affect the ferroptosis pathway. Our findings in brain organoids with PSEN1 mutation complement well the results in human cortical organoids challenged with Aβ that showed an increased expression of ferroptosis-promoting factors like *Phkg243, Txnrd144* and *Pparg42* [50]. Lower levels of CYCS and FTH1 indicate lower mitochondrial content and impaired iron storage, respectively. Higher levels of PCBP1, an iron chaperone protein involved in the ferritin-related iron storage pathway, suggest an increased need to bind iron which could be an indiator of iron overload in the AD-cBOs [51]. In support of these findings, previously it has been shown that FAC-induced iron overload in *Pink−/*− cells resulted in upregulation of MAP1LC3A protein levels [52]. This suggests that the similar pattern of MAP1LC3A increase in AD compared to Iso-cBOs could be an indicator of increased iron level, potentially being related to increased ferroptosis vulnerability.

Interestingly, the presence of functional microglia in brain slices [53] or in human cortical organoids attenuated the Aβ-mediated induction of ferroptosis [50]. This underlines the importance of integrating microglia into the organoid model to mimic the complexity of the brain more accurately [31, 54]. Similarly, astrogliosis is an increasing event during the progression of AD pathology [55]. Our organoid model did not include microglia or astrocytes at day 50, which could be considered a limitation. An additional constraint could arise from the fact that while ferrostatin-1 has also previously shown efficacy in inhibiting ferroptosis in other human organoid models, such as bronchial [56] and gut crypt organoids [57], its utility is hindered by its solubility issues. The strength of our model lies in the development of amyloid beta-like aggregates within human brain AD organoids and effectively demonstrates the preventive action mediated by ferrostatin-1. These findings are in line with our post-mortem results, where we observed variations in the levels of ferroptosis-related proteins between the Aβ plaque and its adjacent regions.

**Fig. 7:**
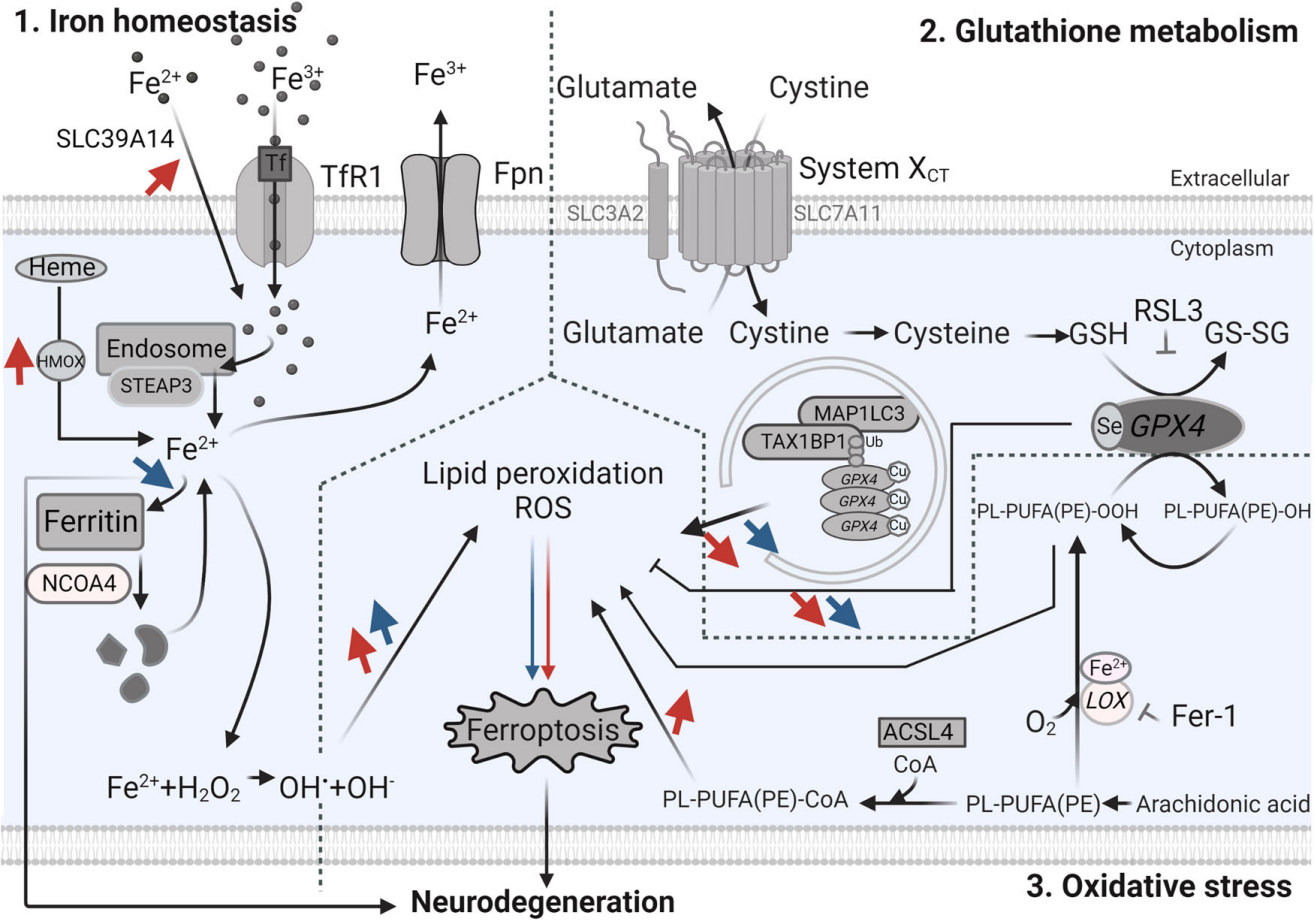
Transcriptomic (red arrows) and proteomic (blue arrows) changes affecting the cellular mechanism related to the ferroptotic pathway. Iron metabolism (left), cysteine and glutathione metabolism (top right), and polyunsaturated fatty acid metabolism (bottom right). ACSL4 Long-chain-fatty acid—CoA ligase 4, Fe^2+^ ferrous iron, Cu Copper, Fe^3+^ Ferric iron, Fer-1 ferrostatin, Fpn Ferroportin, GPX4 glutathione peroxidase 4, GSH glutathione (reduced glutathione form), GS-SG glutathione disulphide (oxidised glutathione form), LOX lipoxygenase, MAP1LC3 microtubule-associated protein 1 light chain 3, NCOA4 nuclear receptor co-activator 4, PL-PUFA(PE)-OH non-toxic polyunsaturated fatty-acids, PL-PUFA(PE)-OOH polyunsaturated fatty acid-containing-phospholipid hydroperoxides, ROS reactive oxygen species, RSL3 (1S,3 R)-RSL3, RTAs radical-trapping antioxidants, Se Selenocysteine, SLC3A2 solute carrier family 3 member 2, SLC39A14 solute carrier family 39 member 14, SLC7A11 solute carrier family 7 member 11, STEAP3 STEAP3 metalloreductasem System XcT, glutamate/cystine antiporter system, TAX1BP1 Tax1 Binding Protein 1, TfR1 transferrin 1 receptor, Ub ubiquitin. Parts of the figure were made with the BioRender programme.

In this study, we integrate various approaches, featuring a robust and well-structured methodology (Fig. 7). We included quantitative analysis of post-mortem data and used a range of various stages of AD severity, to be able to compare early and advanced stages of the pathology. It is important to highlight that while post-mortem results offer valuable insights into the human context, they merely capture a snapshot of ferroptosis-related protein expression changes, allowing for correlations to be drawn. Human brain organoids offer enhanced translatability and add a more intricate simulation of the human brain compared to simpler in vitro cell cultures.

However, the absence of additional cell types, such as microglia, in our organoid model may potentially influence the outcomes. Although the underlying mechanisms responsible for this neuroprotective effect require further investigation, in this study we successfully demonstrate that pathways of ferroptosis and development of Aβ pathology seem to be closely intertwined. While none of these models are flawless on their own, they complement each other and are all essential in comprehensively assessing the connection between ferroptosis and Aβ in AD. Overall our study demonstrated the association between Aβ plaques and the expression of various ferroptosis-related markers in human brain tissue and brain organoids.

## Supplementary information

Supplemental material

## Data Availability

This study data are deposited in GEO under accession number GSE266358.

## Code availability

The code that supports the findings of this study is available from the authors upon request.
