# Supplementary material for: The link between amyloid β and ferroptosis pathway in Alzheimer’s disease progression
Source: Cell Death Dis. 2024 Oct 28;15(10):782. doi: 10.1038/s41419-024-07152-0 (PMC11519607; doi:10.1038/s41419-024-07152-0)
Supplement: Supplementary file 1 — Supplemental material [file 41419_2024_7152_MOESM1_ESM.docx]

**The link between amyloid β and ferroptosis pathway in Alzheimer's progression**

Naďa Majerníková^1,2^, Alejandro Marmolejo-Garza^1,3^, Casandra Salinas Salinas^1^, Minh D.A Luu^1^, Yuequ Zhang^1^, Marina Trombetta-Lima^1,4^, Tamara Tomin^5^, Ruth Birner-Gruenberger^5^, Šárka Lehtonen^6,7^, Jari Koistinaho^7^, Justina C. Wolters^8^, Scott Ayton^9^, Wilfred F.A. den Dunnen^2*^, Amalia M. Dolga^1,2*^

**Supplementary tables**

***Supplementary table 1:*** *List of ferroptosis-related genes.*

**

*Ferroptosis-related genes. ACSL1, Long-chain-fatty-acid—CoA ligase 1; ACSL3, Long-chain-fatty-acid—CoA ligase 3; ACSL4, Long-chain-fatty-acid—CoA ligase 4; ACSL5, Long-chain-fatty-acid—CoA ligase 5; ACSL6, Long-chain-fatty-acid—CoA ligase 6; AIFM2, Apoptosis-inducing factor mitochondria-associated 2; ALOX15, Arachidonate 15-lipoxygenase/15-lipoxygenase-1; ATG5, Autophagy related 5; ATG7, Autophagy related 7; CP, Ceruloplasmin; CYBB, Cytochrome B-245 Beta chain; DHODH, Dihydroorotate dehydrogenase; FTH1, Ferritin heavy chain; FTL, Ferritin light chain; FTMT, Ferritin mitochondrial;* *GCH1, Guanosine triphosphate cyclohydrolase-1; GCLC, Glutamate-cysteine ligase catalytic subunit; GCLM, Glutamate-cysteine ligase modifier subunit; GPX4, Glutathione peroxidase 4, GSS, Glutathione synthetase; HMOX1, Heme oxygenase 1; LPCAT3, Lysophosphatidylcholine acyltransferase 3; MAP1LC3A, Microtubule associated protein 1 light chain 3 Alpha; MAP1LC3B, Microtubule associated protein 1 light chain 3 Beta; MAP1LC3B2, Microtubule associated protein 3 light chain 2 Beta; MAP1LC3C, Microtubule associated protein 1 light chain 3 gamma; NCOA4, Nuclear receptor coactivator 4; PCBP1, Poly(rC)-binding protein 1; PCBP2, Poly(rC)-binding protein 2; PRNP, Prion protein; SAT1, Spermidine/spermine N1-acetyltransferase 1; SAT2, Spermidine/spermine N1-acetyltransferase 2; SLC11A2 Solute carrier family 11 member 2; SLC39A14, Solute carrier family 39 member 14; SLC39A8, Solute carrier family 39 member 8; SLC3A2, Solute carrier family 3 member 2; SLC40A1, Solute carrier family 40 member 1; SLC7A11, Solute carrier family 7 member 11; STEAP3,* STEAP3*Metalloreductase, TF, Transferrin; TFRC, Transferrin receptor; TP53, tumor protein 53; VDAC2, Voltage-dependent anion channel 2; VDAC3, Voltage-dependent anion channel 3. Ferroptosis-related genes were analysed among AD DEGs based on* (23)*.*

***Supplementary table 2:*** *Log2FoldChange of significant genes (padj <0.05) of AD-cBOs compared to Iso-cBOs.*

**

*ACSL6, Long-chain-fatty-acid-CoA ligase 6; FTL, Ferritin light chain; GCLC, Glutamate-cysteine ligase catalytic subunit; GCH1, GTP cyclohydrolase 1; HMOX1, Heme oxygenase 1; MAP1LC3A, Microtubule associated protein 1 light chain 3 Alpha; PCBP2, Poly(rC)-binding protein 2; PRNP, prion protein; SAT1, Spermidine/spermine N1-acetyltransferase 1; SAT2, Spermidine/spermine N1-acetyltransferase 2; SLC39A14, Solute carrier family 39 member 14; SLC39A8, Solute carrier family 39 member 8; TP53, Tumor protein p53; TP53BP1, Tumor protein p53 binding protein, 1.*

***Supplementary table 3: Summarizing table of alterations involving ferroptosis-related proteins and genes across models and methods used.***

*ACSL6;* *Acyl-CoA Synthetase Long Chain Family Member 6, CYCS; Cytochrome c, FTL; ferritin light chain, FTH1; ferritin heavy chain 1, FPN1; ferroportin, GCH1; GTP Cyclohydrolase 1, GCLC; Glutamate-cysteine ligase catalytic subunit, GPX4; Glutathione peroxidase 4, HMOX1; heme oxygenase 1, MAP1LC3A; Microtubule associated protein 1 light chain 3 alpha, NCOA4; nuclear receptor coactivator 4, PCBP1/2; Poly(rC) binding protein 1/2, PRNP; prion protein, SAT1/2; spermidine 1 and 2, SLC3A14/8; Solute carrier family 3 member 14 and 8, TP53; Tumor protein p53, TP53PB1; Tumor protein p53 binding protein 1. Other abbreviations: AD ans Iso cBOs; Alzheimer’s disease and isogenic corticlar brain organoids, Ctr; control, na; not applicable, PM; post mortem, RNA seq, RNA sequencing.*

***Supplementary figure 1: Human-derived PS1-ΔE9 NPCs show significant changes in levels of proteins related to increase ferroptosis resistance.***

***
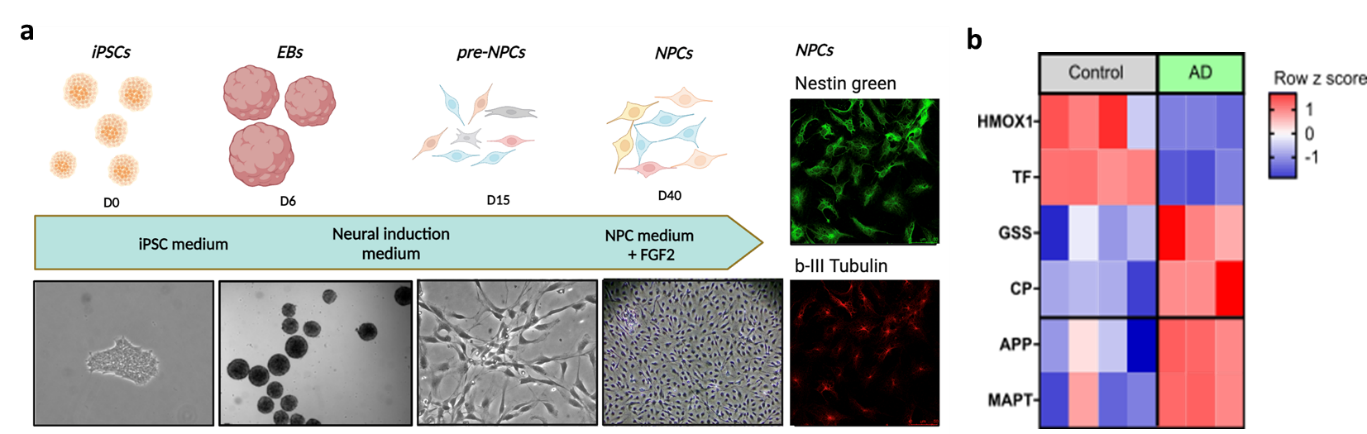
***

*Overview of the neuronal progenitor cell (NPC) generation. NPC expressed nestin and beta 3 tubulin proteins, as visualised by immunofluorescence (a). Heatmap displaying the significantly dysregulated ferroptosis-related proteins in NPCs derived from AD patients compared to controls (padj˂0.05)****.*** *The AD group consisted of three lines from AD patients with the PSEN1-ΔE9 mutation, while the control group included two healthy control lines and two isogenic control lines. This analysis includes the average of three repeated measures per line. In AD-NPCs, an upregulation of proteins associated with ferroptosis resistance, namely Glutathione synthetase (GSS) and ceruloplasmin (CP), was observed. Conversely, proteins linked to increased vulnerability to ferroptosis, such as Heme oxygenase (HMOX1) and Serotransferrin (TF), were downregulated in AD-NPCs compared to the control cell lines. As expected, the amyloid precursor protein (APP) and microtubule-associated protein tau (MAPT), both associated with AD-related pathology, exhibited increased expression in AD compared to control. The results are represented in z scores (b).*

***Supplementary figure 2: Representative example images of ferroptosis-related markers in white (WM) and grey matter (GM).***

*
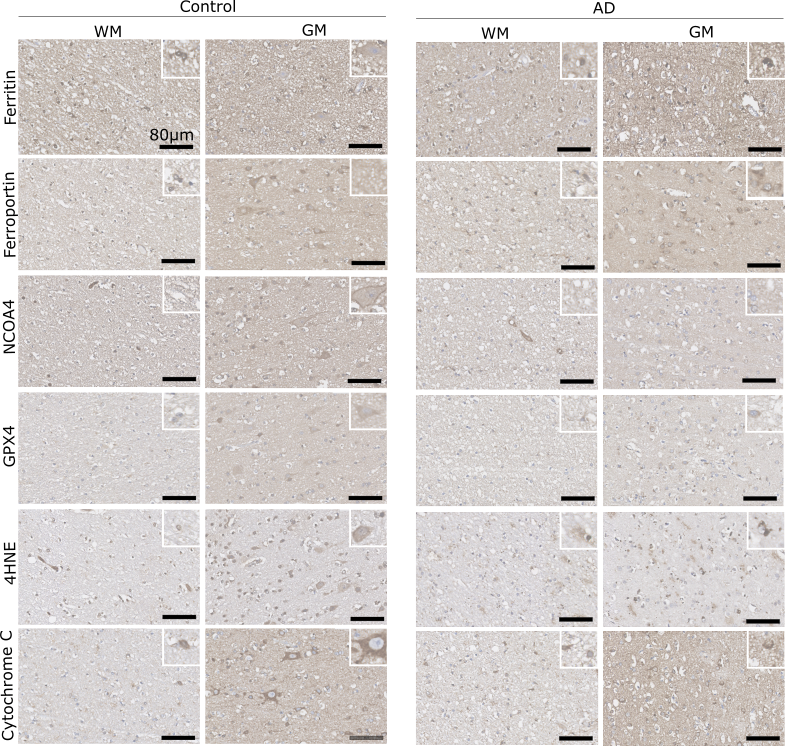
*

*All pictures correspond to 1 example AD subject (Tau stage 6, Aβ stage 5) and 1 example control subject (Tau stage 0, Aβ stage 0). These representative images illustrate the differences between grey (GM) and white matter (WM). Detailed images on the top right corner represent 50x50µm area enlarged two times. For a quantitative evaluation of these differences, please refer to Figure 2. Different cell types (neurons, oligodendrocytes, microglia and astrocytes show different intensities of expression depending on the protein).*

*Differences between the expression of ferroptosis-related markers in the white and grey brain matter: anatomical and cellular distribution*

The analysis of the markers associated with iron homeostasis included ferritin, ferroportin and nuclear receptor co-activator 4 (NCOA4). In the GM of control healthy subjects, ferritin was mainly observed in glial cells and neuronal axons and dendrites, while a weak ferritin signal was observed in smaller, granular neurons. In the WM of control healthy subjects, ferritin signal was mostly detected in glial cells. In the AD brain, while a clear distinction of ferritin signal between GM and WM was not observed, the superficial layers of cortex were more positive compared with deeper cortical layers. The GM of AD brain showed more ferritin positive glial cells in the plaque-related areas with distorted neurites, and glial cells showed more ferritin expression in WM of AD brain than in control healthy subjects.

In both the control subjects and the AD brains, the signal of ferroportin staining was higher in GM than in WM. In the GM of the control subjects, ferroportin was detected in large, pyramidal neurons and localised mostly in the cytoplasm and plasma membrane of the soma, while granular neurons, astrocytes and endothelial cells showed less ferroportin signal. In the WM of the control subjects, ferroportin was mostly detected in the myelin sheets and astrocytes. In the AD brain, similarly as in control subjects, ferroportin was observed in GM in pyramidal neurons and astrocytes. Interestingly, positive areas at different places of the GM were present when surrounded by glial cells, which were not seen in control GM. Since this feature might be related to plaque, we have decided to explore it in more detail and quantitative manner in the following section. Moreover, ferroportin expression in glial cells was strongly expressed in the WM of AD brain compared to WM of control subjects.

The NCOA4 staining showed overall stronger expression in GM compared to WM in both control subjects and AD brain. In the control subjects, NCOA4 was detected in pyramidal neurons, glial and endothelial cells of the GM area. NCOA4 was observed in the cytoplasm of the pyramidal neurons, and at the cell membrane in a punctuate form. In contrast, NCOA4 was detected mostly in astrocytes in the WM of control subjects. In AD, NCOA4 was highly expressed in dendrites of the GM, while the punctuate pattern near cell membrane was much less evident. In the GM of control brain, we obsereved a punctate pattern at the plasma membrane whearas the positive neurons in AD, show more diffused cytoplasmic staining and less punctate membranous staining ***(Supplementary figure 3)***. In WM of AD brain, NCOA4 expression showed a puncuate pattern.

Besides the ferroptosis-related markers associated with iron homeostasis, we next investigated the markers linked to oxidative stress and lipid peroxidation. Two markers related to oxidative stress were included in our study: i) 4-hydroxy-2-nonenal (4HNE), the marker of lipid oxidation, and ii) glutathione peroxidase 4 (GPX4), the most essential antioxidant enzyme involved in ferroptosis. In the GM of control brain, 4HNE staining showed high expression in the cytoplasm of cell bodies of granular neurons and glial cells. Pyramidal neurons were less positive than granular neurons, but some presented punctuate 4HNE staining. In the WM of control brains, 4HNE signal was high in the cell bodies of glial cells. In AD brain, the difference between WM and GM was similar to that in the control brain. However, the number of positive 4HNE puncta seemed to be increased. In WM, 4HNE was mostly observed in glial cells, while granular puncta were less obvious.

The GPX4 staining showed overall higher signal in GM compared to WM. In the GM of control brain, GPX4 expression was observed in pyramidal neurons, while in granular neurons the expression was observed to a lesser extent. In the WM of control brain, GPX4 was detected in axons, and astrocytic glial cells. The WM of AD subjects was slightly positive for GPX4 in glia cells and overall, while in the GM, the GPX4 signal was observed in pyramidal neurons (in the dendritic tree and nucleolus of some neurons) and glial cells.

Cytochrome c, often used as a marker of apoptosis provides information about mitochondrial dysfunction (3). We included cytochrome c in our study, to investigate whether cytochrome c-related mitochondrial damage can be detected in AD brain. In general, we found a higher expression of cytochrome c in GM compared to WM. Cytochrome c was detected in the cytoplasm of both pyramidal and granular neurons and less expressed in glial cells in the GM of control brain, while in the WM cytochrome c was detected in glial cells and to a lesser extent in axons observed as puncta staining. In AD, cytochrome c was less expressed in the neuronal cell bodies compared to control brain. In AD WM, cytochrome c showed a similar pattern as in the WM of control brain. The quantitative evaluation of these differences will be discussed in the following section and depicted in Figure 3.

***Supplementary figure 3: Examples of NCOA4 staining pattern in neurons of the control and AD brain.
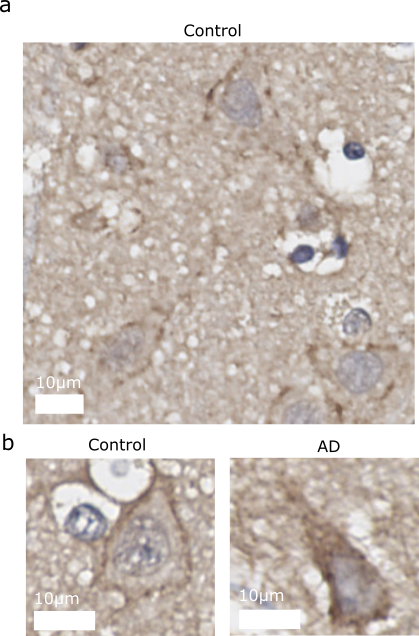
***

*Overview of the NCOA4 staining in the control brain (a) and a detailed view on a single neuron in the control brain showing a punctuate pattern of NCOA4 at the plasma membrane (on the left) and a single neuron in the AD brain depicting translocation of NCOA4 to the cytoplasm (b).*

***Supplementary figure 4:*** ***Positive pixel density scoring of AD-cBOs compared to Iso-cBOs of various ferroptosis-related proteins at day 100 without exclusion of hypoxic cores.***


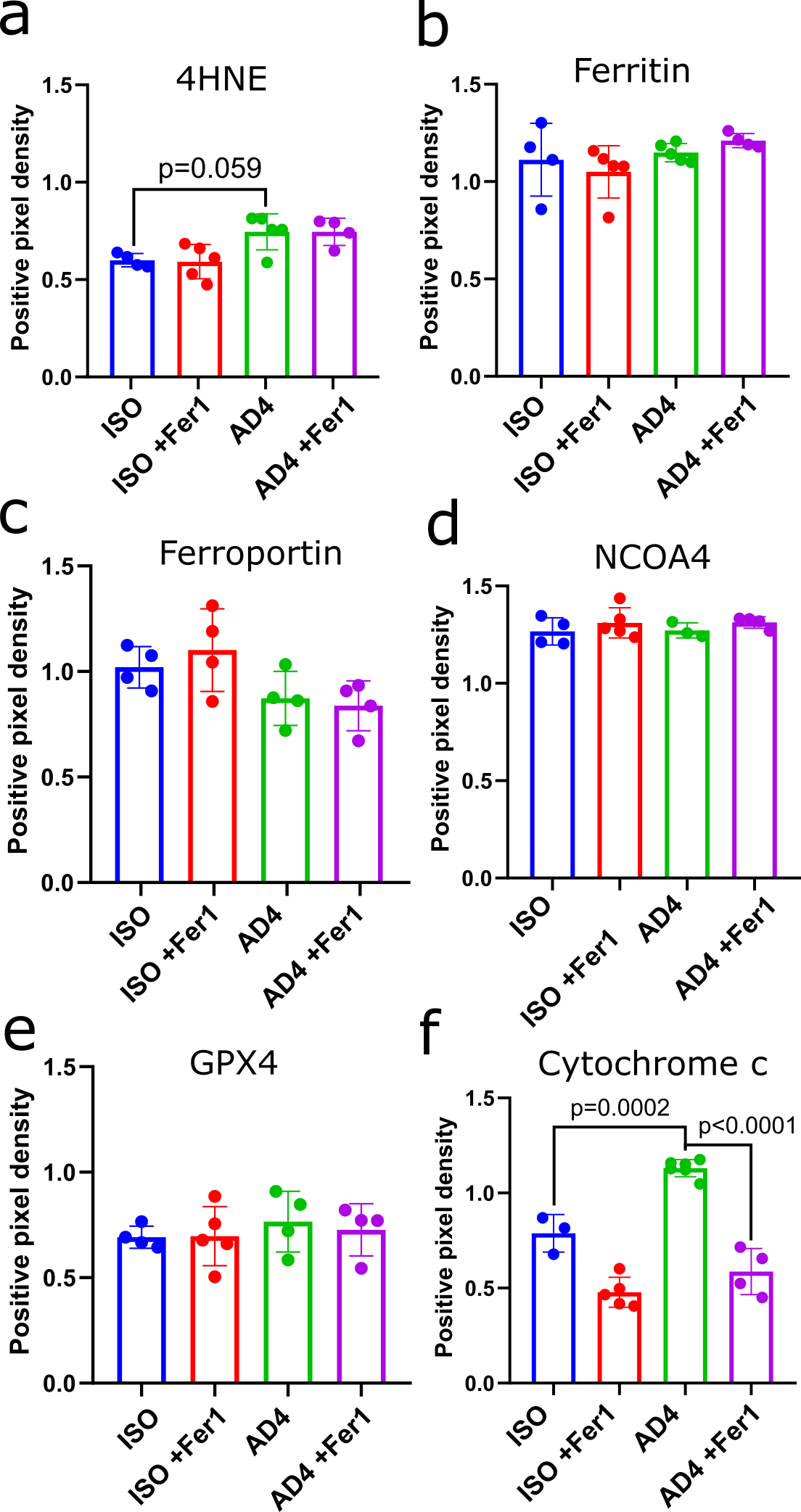


*When selecting the entire organoid for positive pixel density scoring, we observed significantly higher levels of cytochrome c (p=0.0002) in AD-cBOs compared to Iso-cBOs and this was prevented by the pretreatment of fer-1 (p˂0.0001). AD-cBOs at day 100 had higher levels of 4HNE compared to Iso-cBOs (p=0.059), but unlike at day 50, this was not prevented by pretreatment of fer-1. No changes were observed in the levels of other analysed ferroptosis-related proteins when the hypoxic cores of the organoids were included in the analyses. Ordinary one-way ANOVA was used to determine significance (p˂0.05 was considered significant).*

***Supplementary figure 5: Covariate effects on protein expression of ferroptosis-related markers in grey matter of controls and subjects with AD.***


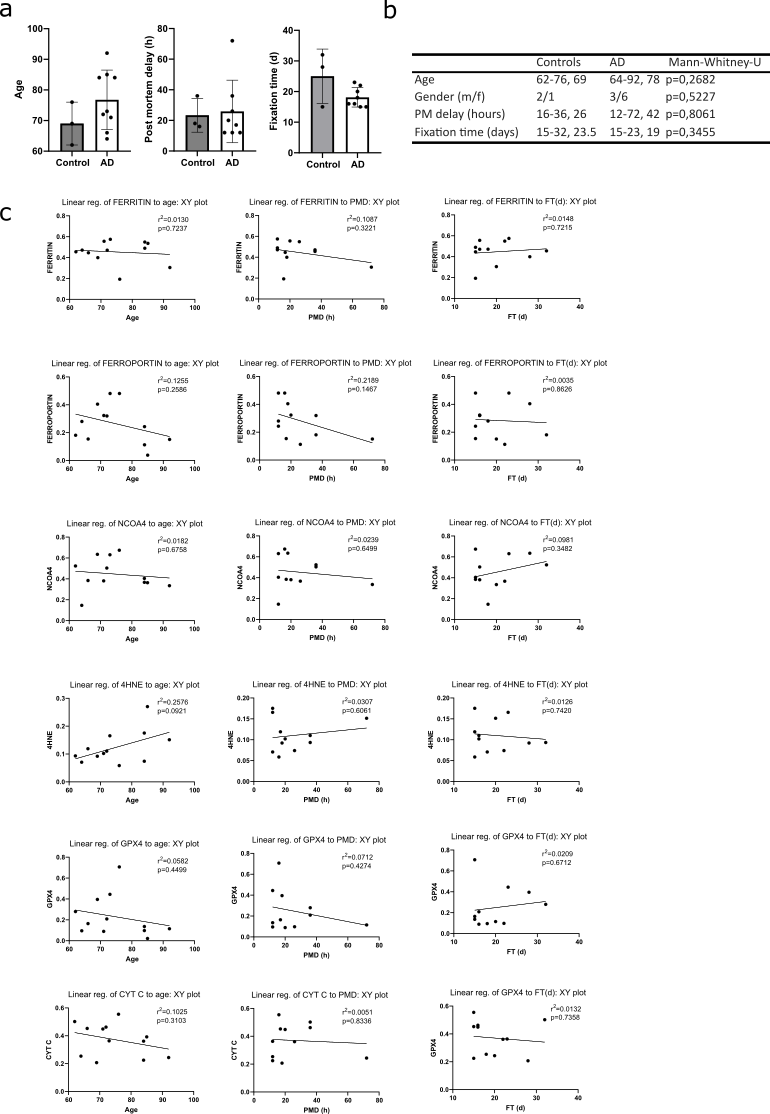


*Differences between controls and subjects with AD in age, post mortem delay (in hours) and fixation time (in days) (a). Table summarizing the range of age, gender (1=male, 2=female), post mortem delay (PMD) and fixation time (FT) between controls and subjects with AD. Numbers in second and third column are reported as: min-max, median. No significant differences were observed between controls and AD subjects (Mann-Whitney U test, p < 0.05) (b). A scatter plot with simple linear regression illustrating the level of correlation between age, post mortem delay (PMD) in hours (h) and fixation time (FT) in days (d) and each ferroptosis-related marker expression, along with the line of best fit. No significant correlation between the ferroptosis-related markers' expressions with any of the studied variables has been observed (Pearson's correlation test and simple linear regression, r^2^ <0.6000, p < 0.05) (c).*
